# Anti-VEGF vascular remodeling drives germinal center B cell-rich tertiary lymphoid structures during antibody-toxin and anti-CD40 combination therapy in glioblastoma

**DOI:** 10.21203/rs.3.rs-9350019/v1

**Published:** 2026-04-17

**Authors:** Anjali Barnwal, Weixuan Yan, Sarah L. Cook, Thomas Urup, Kevin Stevenson, Yiping He, Cristina Osorio, Scott Parker, Edwige Edouard, Michelle Bowie, Hunter C. Jackson, Roger E. McLendon, Annick Desjardins, David M. Ashley, Darell D. Bigner, Vidyalakshmi Chandramohan

**Affiliations:** 1 The Preston Robert Tisch Brain Tumor Center, Duke University Medical Center, Durham, NC, USA.; 2 Department of Neurosurgery, Duke University Medical Center, Durham, NC, USA.; 3 Danish Comprehensive Cancer Center, Brain Tumor Center (DCCC-BTC), Blegdamsvej 9, Copenhagen, 2100, Denmark.; 4 Department of Oncology, Rigshospitalet, Blegdamsvej 9, Copenhagen, 2100, Denmark.; 5 Molecular Physiology Institute, Duke University Medical Center, Durham, NC, USA.; 6 Department of Pathology, Duke University Medical Center, Durham, NC, USA.

## Abstract

Glioblastoma (GBM) is characterized by aberrant neovascularization and angiogenesis. Bevacizumab (Avastin), a monoclonal antibody targeting vascular endothelial growth factor (VEGF), is clinically used with chemotherapy for patients with GBM, yet its impact on the tumor immune microenvironment remains poorly understood. Transcriptomic profiling of matched pre- and post-treatment tumors from patients with recurrent GBM treated with bevacizumab plus the topoisomerase inhibitor irinotecan revealed significant immune reprogramming in responders, with post-treatment tumors displaying features indicative of tertiary lymphoid structure (TLS) formation and enhanced antitumor immunity. In preclinical orthotopic glioma models, combining αVEGF therapy with intratumoral epidermal growth factor receptor (EGFR)-targeted cytotoxic therapy and αCD40 immunotherapy normalized the tumor vasculature, promoted lymphatic vessel growth, induced tumor cell killing, increased intratumoral T cells, plasma cells, germinal center B cells, and antigen-presenting and tissue-repair-associated macrophages, while reducing immunosuppression and supporting mature TLS formation with an antitumor immune phenotype. Functionally, the tumor-, immune-, and vascular-targeted combination therapy significantly improved tumor control, extended survival, and induced durable antitumor memory in glioma models. Collectively, these results identify VEGF as a central regulator of the vascular-immune axis during cytotoxic EGFR+αCD40 therapy in GBM and provide a strong rationale for combining vascular-, tumor-, and immune-targeted approaches to overcome therapeutic resistance and improve outcomes for patients with GBM.

## Introduction

Glioblastoma (GBM) is the most common and aggressive primary brain tumor in adults, with a median survival of approximately 8–12 months and a near 100% mortality rate^1^. While immunotherapies have been successful in multiple cancer types, GBM remains refractory^2^. The aggressive progression, high recurrence, and therapy resistance in GBM are driven by extensive neovascularization, intratumoral heterogeneity, and a profoundly immunosuppressive microenvironment^3^. The glioma vasculature is structurally and functionally abnormal, resulting in hypoxia, impaired immune cell trafficking, and restricted access of therapeutic agents to the tumor^4–6^. Consequently, GBM remains largely unresponsive to immune checkpoint blockade and other immune-based interventions, highlighting the need for therapeutic strategies that target tumor cells while simultaneously remodeling the abnormal tumor vasculature and immunosuppressive tumor microenvironment.

Vascular endothelial growth factor (VEGF), released by most solid tumors, including GBM, is a pro-angiogenic factor that induces the formation of tumor neovasculature^7^. Targeting VEGF has been a central approach to managing tumor-associated angiogenesis^8–10^. Bevacizumab (Avastin), a monoclonal antibody against VEGF, is approved for the treatment of recurrent GBM (rGBM) and has been shown to transiently normalize tumor vasculature, reduce edema, and improve progression-free survival when combined with chemotherapy^11,12^. However, its impact on overall survival remains limited, and the immunological consequences of VEGF blockade in GBM are poorly understood. Beyond its role in angiogenesis, VEGF exerts potent immunosuppressive effects by impairing dendritic cell maturation, inhibiting T-cell activation, supporting the expansion of regulatory T cells and myeloid-derived suppressor cells, and promoting tumor-associated macrophage infiltration^13–15^. Recent studies have suggested that vascular normalization may create a permissive environment for productive immune activation in solid tumors^13,14^. In particular, normalization of the tumor vasculature can enhance antigen presentation, increase lymphocyte trafficking, and support the formation of tertiary lymphoid structures (TLS)—organized aggregates of B cells, T cells, and antigen-presenting cells that resemble secondary lymphoid organs^16,17^. TLS serve as intratumoral immune niches that sustain antigen-specific B- and T-cell responses and have been associated with improved prognosis and response to immunotherapy in several cancers^16,18–20^. Thus, αVEGF therapy has the potential to reshape the glioma immune microenvironment, not only by normalizing vasculature but also by alleviating immune suppression, facilitating immune cell infiltration, promoting TLS formation, and augmenting anti-GBM immunity.

Herein, using publicly available bulk transcriptomic data^21^, we found that treatment with Avastin plus the topoisomerase inhibitor irinotecan decreases endothelial cells and increases immune cell subsets associated with TLS formation, and these changes are associated with improved survival in patients with rGBM. We previously demonstrated that intratumoral delivery of a bispecific antibody-toxin conjugate (ATC) targeting wild-type epidermal growth factor receptor (EGFRwt) and mutant EGFR variant III (EGFRvIII), combined with αCD40 agonist antibody (ATC+αCD40; A+C), elicits potent antitumor immunity and durable tumor regression in murine glioma models^22^. ATC induces tumor cell death and antigen release, while αCD40 promotes a proinflammatory phenotype in tumor-associated macrophages (TAMs) and tumor-specific CD8^+^ T cell immunity, together generating an antitumorigenic immune milieu^22^. Given that aberrant neovascularization and angiogenesis are characteristic features of GBM, we hypothesized that VEGF blockade and vascular normalization could amplify the immunostimulatory potential of A+C therapy by promoting the formation of TLS-like structures within the GBM microenvironment. Consistent with our hypothesis, in orthotopic glioma models, VEGF blockade (αVEGF [V]) when combined with A+C induced vascular normalization and lymphatic vessel growth. Single-cell RNA sequencing analysis of tumor samples from the A+C+V treatment group demonstrated a transcriptional profile consistent with activated T cells, plasma cells, germinal center B cells, and proinflammatory myeloid cells, supporting the formation of mature TLS and decreased immunosuppression. Further, immunohistochemistry and multiplex immunofluorescence analyses confirmed CD4^+^ T cell and B cell infiltration, as well as TLS formation, at the tumor periphery. Finally, αVEGF therapy potentiated A+C antitumor immunity, resulting in improved survival and durable long-term memory response. Our findings reveal a previously unrecognized immunomodulatory and antitumorigenic role for A+C+V therapy and provide a strong rationale for combining tumor-killing and immune-activation with vascular normalizing agents to overcome therapy resistance in GBM.

## Results

### T follicular helper (Tfh) cells and plasma cells are markers of response to the bevacizumab-chemotherapy combination in rGBM patients.

**Figure 1.**
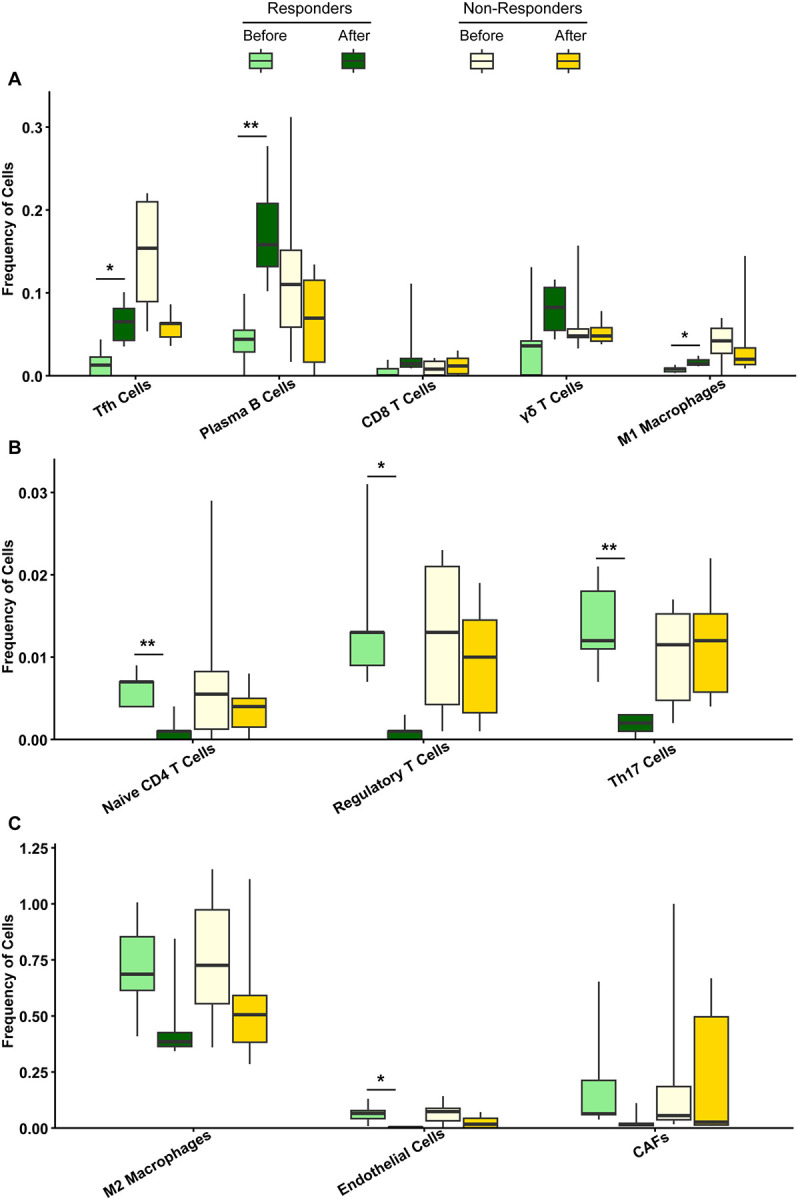
The combination of bevacizumab and chemotherapy promotes TLS formation in the GBM TME of responders. Box plots showing the estimated cell-type proportions from TIMER3 deconvolution of pre-treatment (n=20) and post-treatment (n=16) tumor transcriptomic data from responder (n=6) vs non-responder (n=10) rGBM patients treated with a combination of bevacizumab and chemotherapy. (**A**) Box plots of Tfh cells, plasma B cells, CD8^+^ T cells, γδ T cells, and M1 macrophages. (**B**) Box plots of naïve CD4^+^ T cells, regulatory T cells, and Th17 cells. (**C**) Box plots of M2 macrophages, endothelial cells, and CAFs. Box plots display the median (center line) with the interquartile range (IQR). Statistical analysis was performed using an unpaired t-test. *p<0.05, **p<0.01.

Bevacizumab (αVEGF) and irinotecan or lomustine improve progression-free survival in approximately 30% of patients with rGBM^21^. Gene expression analysis demonstrated decreased angiogenesis and increased neuronal development and signaling in post-treatment tumor tissue from rGBM patients who responded to bevacizumab plus irinotecan^21^. However, the impact of the combination therapy on the glioma immune compartment remains unknown. To investigate this, we performed immune deconvolution analysis on publicly available bulk RNA-sequencing data from 36 rGBM patients (20 pre-bevacizumab and 16 post-bevacizumab samples, including 16 paired and 4 unpaired samples). Among the 16 paired samples, 6 were from patients classified as responders, and 10 were from patients classified as non-responders^21^. Immune decovolution analysis showed a significant increase in antitumorigenic immune cells, including Tfh cells, plasma B cells, CD8^+^ T cells, γδ T cells, and M1 macrophages in post-treatment tumor tissue from responders (Figure 1A). In contrast, immunosuppressive protumorigenic subsets, such as naïve T cells, regulatory T cells, T helper 17 (Th17) cells^23^, M2 macrophages, endothelial cells, and cancer-associated fibroblasts (CAFs) were significantly reduced post-treatment, specifically in responders (Figures 1B–1C). Importantly, pre- and post-treatment analysis of responders and non-responders combined failed to reveal this distinction in the GBM microenvironment, underscoring the need for outcome-based biomarker analysis to improve therapy response (**Supplemental Figures 1A-1C**).

Among the most significantly altered populations post-bevacizumab combination treatment were Tfh cells and plasma B cells, which are central drivers of TLS formation^24^. Tfh cells secrete CXCL13, a chemokine that recruits and activates B cells, leading to the development of mature TLS^25,26^. A reduction in Treg levels was also observed in responders, which is commonly associated with enhanced proliferating lymphocytes in TLS^27^. Taken together, these data suggest that the bevacizumab-chemotherapy combination overcomes immunosuppression, promotes TLS formation, and remodels the GBM immune landscape in favor of antitumor activity, contributing to treatment response in a subset of rGBM patients.

### αVEGF-induced vascular remodeling promotes ATC+αCD40 antitumor immunity in GBM

**Figure 2.**
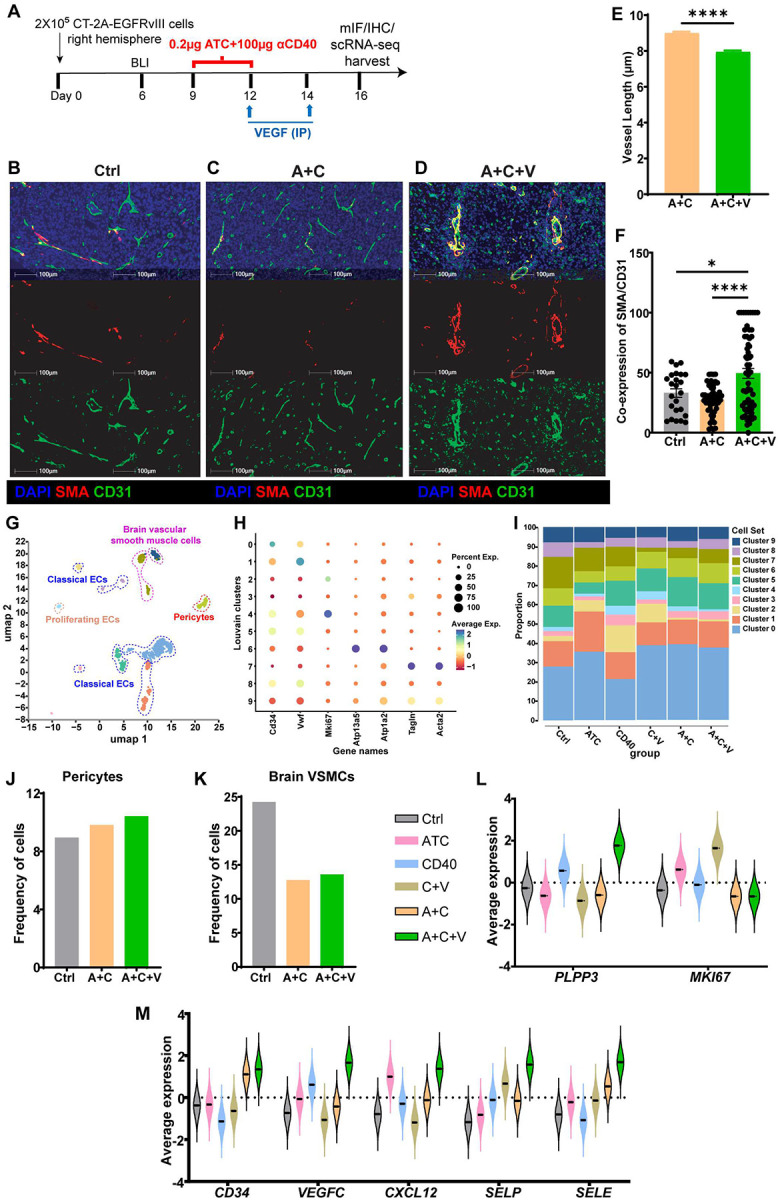
αVEGF post ATC and αCD40 combination promotes vascular remodeling in CT-2A-EGFRvIII glioma-bearing mice. (**A**) Study outline for the treatment of CT-2A-EGFRvIII tumors in C57BL/6J female mice. CT-2A-EGFRvIII tumors were treated from days 9 to 12 post-implantation by CED with Control (Ctrl), ATC, αCD40, C+αVEGF (C+V), A+C, or A+C+V therapies (n=4–5 per group). On day 16 post-tumor implantation, tumors were harvested for multiplex immunofluorescence (mIF) or immunohistochemistry (IHC) analysis. (**B**-**D**) Representative mIF images of tumor sections from CT-2A-EGFRvIII tumors treated with (**B**) Ctrl, (**C**) A+C, or (**D**) A+C+V, stained for DAPI (4′,6-diamidino-2-phenylindole) (nucleus, blue), SMA (red), and CD31 (green). (**E**) Bar graph showing the vessel length (n=30780 vessels/group) quantified from IHC images of CT-2A-EGFRvIII tumors (Supplementary Figure 2). (**F**) Bar graph showing the ratio of SMA to CD31 (pericyte coverage of vascular endothelium) in the brains of CT-2A-EGFRvIII tumor-bearing mice, quantified from IHC images (Supplementary Figure 2). (**G**) UMAP representation of different endothelial cell clusters in CT-2A-EGFRvIII tumors treated with Ctrl, ATC, αCD40, C+V, A+C, or A+C+V. A total of 897 endothelial cells across all treatment groups were subsetted from the scRNA-seq dataset (41,439 cells in total). (**H**) Dot plot showing average expression of cluster-specific marker genes in CT-2A-EGFRvIII tumors treated with Ctrl, ATC, αCD40, C+V, A+C, or A+C+V. (**I**) Bar graph showing the percentage of different endothelial cell clusters in CT-2A-EGFRvIII tumors treated with Ctrl, ATC, αCD40, C+V, A+C, or A+C+V. (**J**-**K**) Bar graphs showing the frequency of (**J**) pericytes and (**K**) VSMCs in CT-2A-EGFRvIII tumors treated with Ctrl, A+C, or A+C+V. (**L**) Violin plot showing the average expression of *PLPP3* and *MKI67* in CT-2A-EGFRvIII tumors treated with Ctrl, ATC, αCD40, C+V, A+C, or A+C+V. (**M**) Violin plot showing the average expression of *CD34*, *VEGFC*, *CXCL12*, *SELP*, or *SELE* in CT-2A-EGFRvIII tumors treated with Ctrl, ATC, αCD40, C+V, A+C, or A+C+V. Data represented as mean ± SEM. Data is from one experiment. Statistical analysis was performed using 2-way ANOVA. *p<0.05, ****p<0.0001.

We previously demonstrated that intratumoral administration of the ATC and αCD40 combination (A+C) by convection-enhanced delivery (CED) induces CD8^+^ T cell infiltration and confers long-term survival in glioma-bearing mice^22^. For antigen-specific recognition and elimination of tumor cells, cytotoxic CD8^+^ T cells need to extravasate across the vascular endothelium and into the tumor microenvironment (TME)^28^. Aberrant tumor vasculature characterized by a leaky network of immature microvessels with reduced pericyte coverage^29^ presents a major impediment to CD8^+^ T cell infiltration and therefore might impair A+C therapeutic response^30^. VEGF inhibitors have been shown to repair and normalize the tumor vasculature and improve immunotherapy efficacy by inducing T cell recruitment into the TME^17,31–33^. Therefore, we investigated whether systemic αVEGF (V) administration following A+C CED induces vascular normalization in the CT-2A-EGFRvIII orthotopic glioma model. We used CD31 as a marker of endothelial cells to detect alterations in vascular morphology and smooth muscle actin (SMA) to assess pericyte coverage of the vascular endothelium^34^. Previous studies have shown that VEGF blockade-mediated vascular normalization is optimal between days 2–5 post-treatment and synergizes with radiation therapy in brain tumor models^35^. Hence, coronal sections of CT-2A-EGFRvIII tumor-bearing hemispheres from control, A+C, and A+C+V treatment groups were examined for CD31 and SMA expression by immunohistochemistry (IHC) and multiplex immunofluorescence (mIF) analysis at four days post A+C+V therapy (Figure 2A). Both mIF and IHC analyses demonstrated increased pericyte coverage of endothelial vessels in the A+C+V group compared with the A+C and control groups (Figures 2B–2D and **Supplemental Figures 2A-2C**), suggesting a transition toward a more mature and functionally organized vascular network^36^. Further, quantitative analysis of CD31^+^ endothelial vessel length from IHC images demonstrated a significant decrease in the A+C+V group compared to the A+C group (P<0.0001) (Figure 2E and **Supplemental Figures 2D-2E**). Consistent with enhanced pericyte coverage post VEGF blockade, the ratio of SMA to CD31 (pericyte coverage of vascular endothelium) was significantly higher in the brains of A+C+V treated mice compared to both A+C (P<0.0001) and control (P=0.014) treated mice (Figure 2F and **Supplemental Figures 2D-2E**).

Next, to decipher A+C+V therapy-mediated changes in the GBM TME, we performed single-cell RNA sequencing (scRNA-seq) analysis of CT-2A-EGFRvIII tumors (n=3–4 mice/group) treated with control, ATC, αCD40, ATC+αCD40 (A+C), αCD40+αVEGF (C+V), and ATC+αCD40+αVEGF (A+C+V). Following stringent quality control, a total of 41,439 single cells were retained for downstream analysis. Unsupervised clustering coupled with uniform manifold approximation and projection (UMAP) visualization revealed a diverse cellular landscape composed of six major clusters. These included tumor (clusters 21 and 24; *EGFR*), endothelial (ECs; clusters 16–18; *CD34*), myeloid (clusters 2, 4, 8, 9, 11, and 12; *AIF1*, *CX3CR1*, and *TMEM119*), natural killer (NK) (cluster 14; *NKG7*), B (clusters 3, 10, and 15; *CD19* and *CD22*), and T (clusters 6 and 7; *CD4*, *CD8A*, and *CD8B*) cell clusters, as shown in **Supplemental Figures 3A** and **3B**. Notably, both A+C and A+C+V treatments displayed a distinct cellular composition compared to all other treatment groups (**Supplemental Figure 3C**). Specifically, tumors from mice treated with A+C or A+C+V showed the greatest enrichment of both B and T cells, indicating a markedly enhanced adaptive immune response (**Supplemental Figures 3C** and **3D**). In contrast, the proportions of tumor and NK cells were reduced in A+C and A+C+V groups relative to the remaining cohorts (**Supplemental Figures 3C** and **3E**), potentially indicating improved tumor control and a reprogramming of NK cell immunosuppression in the GBM microenvironment. The overall frequencies of endothelial and myeloid cells remained largely consistent across all treatment groups, suggesting that A+C and A+C+V therapies reprogram the transcriptional and functional states of these cells rather than their abundance (**Supplemental Figures 3C** and **3F**).

To characterize therapy-induced vascular changes in greater detail, we subsetted and reclustered *CD45*^−^*CD34*^+^ endothelial cells (N=897 single cells) into ten transcriptionally distinct clusters, encompassing classical endothelial cells (clusters 0, 1, 2, 3, 5, and 8; *CD34* and von Willebrand factor [*VWF*]), proliferating endothelial cells (cluster 4, *MKI67*), pericytes (cluster 6; *ATP13A5* and *ATP1A2*), and brain vascular smooth muscle cells (VSMCs) (clusters 7 and 9; *TAGLN* and *ACTA2*), underscoring the structural complexity of GBM vasculature (Figures 2G and 2H). Interestingly, the A+C+V treatment group showed an increase in pericyte abundance (Figures 2I [cluster 6] and **2J**) along with a concomitant reduction in the frequency of brain vascular smooth muscle cells (VSMCs) (Figures 2I [clusters 7 and 9] and 2K). Previous studies have shown that VSMCs are elevated in GBM relative to normal neural tissue and often expand in association with pathological neovascularization^37^. In contrast, pericytes play a critical role in vessel stabilization, a process increasingly recognized as therapeutically advantageous compared with simple vessel depletion during vascular normalization in glioma^38,39^. Additionally, *PLPP3* (Lipid Phosphate Phosphatase 3), an integral plasma membrane protein involved in the dephosphorylation of lipid substrates such as sphingosine-1-phosphate (S1P) and lysophosphatidic acid (LPA)^40^, and promotes vascular integrity and proper function (normalization) of endothelial cells, was highly expressed, specifically in the A+C+V group (Figure 2L). In contrast, expression of *MKI67*, a marker of microvessel proliferation^41^ and tumor aggressiveness, was the lowest in the A+C+V group (Figure 2L). Therefore, the observed shift toward a *PLPP3*-high, *MKI67*-low, pericyte-enriched, and VSMC-depleted vascular niche in the A+C+V group suggests a non-proliferating, normalized, structurally supported vasculature that is better equipped to facilitate effective antitumor immune responses.

Moreover, *CD34* and *VEGFC* expression levels were elevated in the A+C+V group compared with all other treatment arms (Figure 2M). CD34^+^ endothelial cells support the maturation and stabilization of lymphatic vessels, a process known to reduce tumor immune evasion by enhancing antigen drainage and immune cell trafficking^42^. Meanwhile, VEGF-C has been shown to enhance CD8^+^ T-cell priming in deep cervical lymph nodes, promote T cell infiltration into tumor tissue, and accelerate GBM clearance^43^. Further, chemokines and adhesion molecules associated with leukocyte transmigration across the blood-brain barrier (BBB), including *CXCL12*^44^, *SELP*, and *SELE*^45^ were upregulated in the A+C+V group compared to other groups (Figure 2M). Collectively, these results suggest that A+C+V therapy promotes vascular remodeling conducive to increased intratumoral immune cell infiltration.

### A+C+V therapy increases T follicular helper memory (TFHM) CD4^+^ T cells in the GBM TME

**Figure 3.**
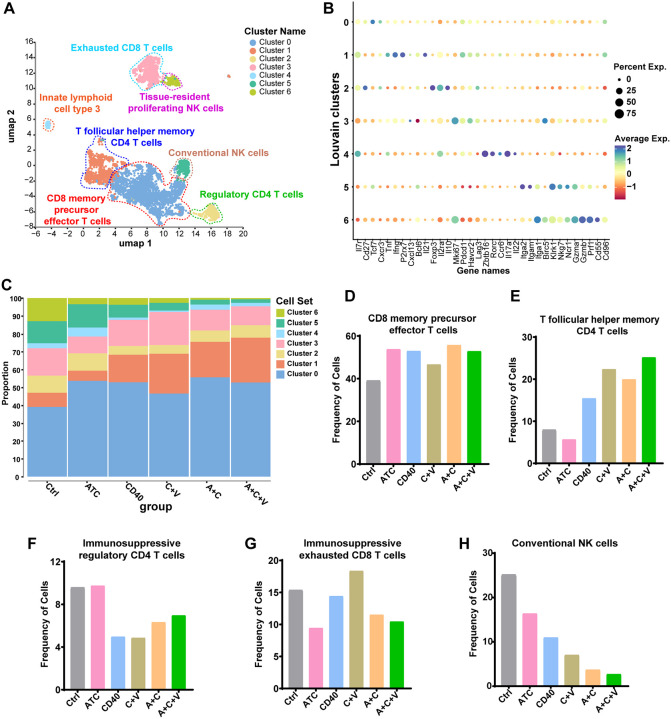
ATC+αCD40+αVEGF therapy induces T follicular helper memory CD4^+^ T cells. (**A**) UMAP representation of different T cell clusters in CT-2A-EGFRvIII tumors treated with Ctrl, ATC, αCD40, C+V, A+C, or A+C+V. A total of 5,418 T cells across all treatment groups were subsetted from the scRNA-seq dataset (41,439 cells in total). (**B**) Dot plot showing average expression of cluster-specific marker genes in CT-2A-EGFRvIII tumors treated with Ctrl, ATC, αCD40, C+V, A+C, or A+C+V. (**C**) Bar graph showing the percentage of different T cell clusters in CT-2A-EGFRvIII tumors treated with Ctrl, ATC, αCD40, C+V, A+C, or A+C+V. (**D**-**H**) Bar graphs showing the frequency of (**D**) CD8^+^ memory precursor effector T cells, (**E**) T follicular helper memory CD4^+^ T cells, (**F**) regulatory CD4^+^ T cells, (**G**) immunosuppressive exhausted CD8^+^ T cells, and (**H**) conventional NK cells in CT-2A-EGFRvIII tumors treated with Ctrl, ATC, αCD40, C+V, A+C, or A+C+V.

We next investigated the impact of A+C+V therapy-induced vascular remodeling on lymphocyte infiltration into the GBM TME. Subsetting, reclustering, and UMAP analysis of *CD45*^+^*CD3*^+^ tumor infiltrating lymphocytes (TILs) (N=5,418 single cells) identified seven transcriptionally distinct single-cell clusters (Figure 3A). Among these, cluster 0 was identified as cytokine producing CD8 memory precursor effector T cells (MPEC)^46^ (*IL7R, CD27, TCF7, CXCR3, TNF*, and *IFNG*), cluster 1 as TFHM^46^ CD4^+^ T cells (*P2RX7, CXCL13, BCL6, IL21*, and *IFNG*), cluster 2 as regulatory CD4^+^ T cells (*FOXP3, IL2RA*, and *IL10*) and cluster 3 as proliferating immunosuppressive exhausted CD8^+^ T cells (*MKI67, PDCD1, HAVCR2*, and *LAG3*). Cluster 4 corresponded to natural cytotoxicity receptor negative (NCR-) innate lymphoid cell type 3^47,48^ (ILC3; *ZBTB16, RORC*, *CCR6*, *IL17A*, and *IL22*) while clusters 5 and 6 consisted of conventional (*ITGA2* and *ITGAM*) and tissue-resident proliferating (*ITGA1, MKI67*, and *BIRC5*) NK cells (*KLRK1, NKG7, NCR1, GZMA, GZMB*, and *PRF1*)^49^ (Figures 3A and 3B). Compared to the control group, scRNA-seq analysis revealed an increase in the proportion of MPEC CD8^+^ T cells across all treatment groups (Figures 3C [cluster 0] and 3D). However, we noted the highest increase in TFHM CD4^+^ T cells in the A+C+V group (25%) (Figures 3C [cluster 1] and 3E). Specifically, IFN-γ-producing Tfh cells contribute to pulmonary protection by promoting the differentiation of lung-resident memory B cells into plasma cells^50^. On the contrary, the highest proportion of immunosuppressive CD4^+^ regulatory T cells was observed in the control (10%) and ATC monotherapy (10%) groups (Figures 3C [cluster 2] and 3F). Similarly, immunosuppressive exhausted CD8^+^ T cells were highest (~14–19%) in the control, αCD40, and C+V groups and lowest (~9–12%) in the ATC, A+C, and A+C+V groups (Figures 3C [cluster 3] and 3G). Interestingly, NK cells (clusters 5 + 6) (Figures 3C and 3H) constituted the highest proportion (~25%) of infiltrating immune cells in the control group and the lowest proportion (~2–3%) in the A+C and A+C+V groups. We hypothesize that conventional NK cells (*ITGA2* and *ITGAM*) from the circulation are recruited and reprogrammed into protumorigenic CCL5-low, CD96-high tissue-resident NK cells (ITGA1) in the GBM TME^49^, and that A+C and A+C+V therapies overcome GBM TME immunosuppression. Together, these findings support a role for VEGF blockade in polarizing A+C antitumor immunity toward a TFHM CD4^+^ T cell response favorable for TLS formation and memory B cell differentiation^25,51^ in the GBM TME.

### αCD40 activates humoral immunity in the GBM TME

**Figure 4.**
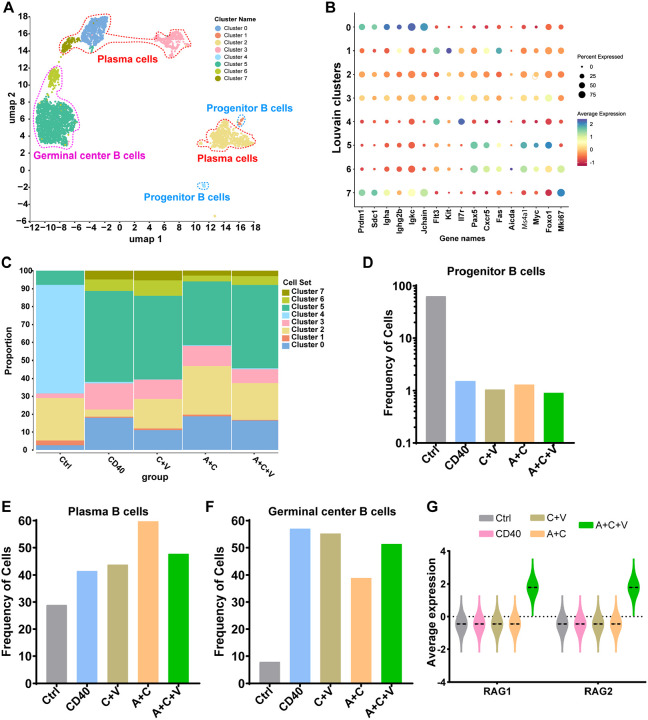
αCD40 promotes B cell activation in CT-2A-EGFRvIII glioma-bearing mice. (**A**) UMAP representation of different B cell clusters in CT-2A-EGFRvIII tumors treated with Ctrl, αCD40, C+V, A+C, or A+C+V. A total of 3,685 B cells across all treatment groups were subsetted from the scRNA-seq dataset (41,439 cells in total). (**B**) Dot plot showing average expression of cluster-specific marker genes in CT-2A-EGFRvIII tumors treated with Ctrl, αCD40, C+V, A+C, or A+C+V. (**C**) Bar graph showing the percentage of different B cell clusters in CT-2A-EGFRvIII tumors treated with Ctrl, αCD40, C+V, A+C, or A+C+V. (**D**-**F**) Bar graphs showing the frequency of (**D**) progenitor B cells, (**E**) plasma B cells, and (**F**) germinal center B cells in CT-2A-EGFRvIII tumors post indicated treatments. (**G**) Violin plot showing the average expression of *RAG1* and *RAG2* in CT-2A-EGFRvIII tumors post indicated treatments.

Next, we investigated ATC, αCD40, and αVEGF mono- and combination-therapy-mediated changes in the B cell compartment by subclustering B cells (N=3,685 single cells). The ATC monotherapy group had very few B cells and was excluded from the analysis. UMAP analysis identified three major B cell subtypes (Figure 4A and 4B): progenitor B cells (FLT3, KIT, IL7R; clusters 1 and 4), plasma cells (PC) (*PRDM1, SDC1, IGHA, IGHG2B, IGKC*, and *JCHAIN*; clusters 0, 2, 3, and 7), and germinal center B (GCB) cells (*PAX5, CXCR5, FAS, AICDA, MS4A1, MYC*, and *FOXO1;* clusters 5 and 6). In the control group, the B cell compartment (~63%) was dominated by progenitor B cells, indicating minimal activation and differentiation (Figure 4C [clusters 1 and 4] and 4D). In contrast, both A+C (1.3%) and A+C+V (0.9%) treatments had markedly reduced proportion of progenitor B cells (clusters 1 and 4), and an increased proportion of PC (A+C: 60%) (Figure 4C [clusters 0, 2, 3, and 7] and 4E) and GCB cells (A+C+V: 51%) (Figure 4C [clusters 5 and 6] and 4F), respectively, suggesting that while A+C induces B cell activation, differentiation, and antibody production, αVEGF-mediated vascular remodeling supports germinal center formation (Figure 4F). The GCB cells were higher in αCD40 (57%), C+V (55%), and A+C+V (51%) groups, confirming a role for CD40 signaling in GCB cell survival^52^. Moreover, *RAG1/2* expression on B cells was significantly higher in the A+C+V group compared to all the other groups, suggesting immunoglobulin gene rearrangements and affinity maturation in activated B cells within the GC^53–55^ (Figure 4G). Further, the proliferation marker *MKI67* was highest in clusters 6 and 7 (Figure 4B), suggesting that these clusters consist of proliferating centroblasts (9%) and plasmablasts (5%), which were highest in the C+V group (Figure 4C). While αCD40, A+C, and A+C+V treatments exhibited high levels of GCB cells, given the abundance of TFHM cells (Figure 3E) and vascular remodeling (Figure 2) in the A+C+V group, we hypothesize that the GBM TME is more favorable to TLS formation following A+C+V therapy.

### αVEGF treatment promotes the formation of TLS in a preclinical glioma model

**Figure 5.**
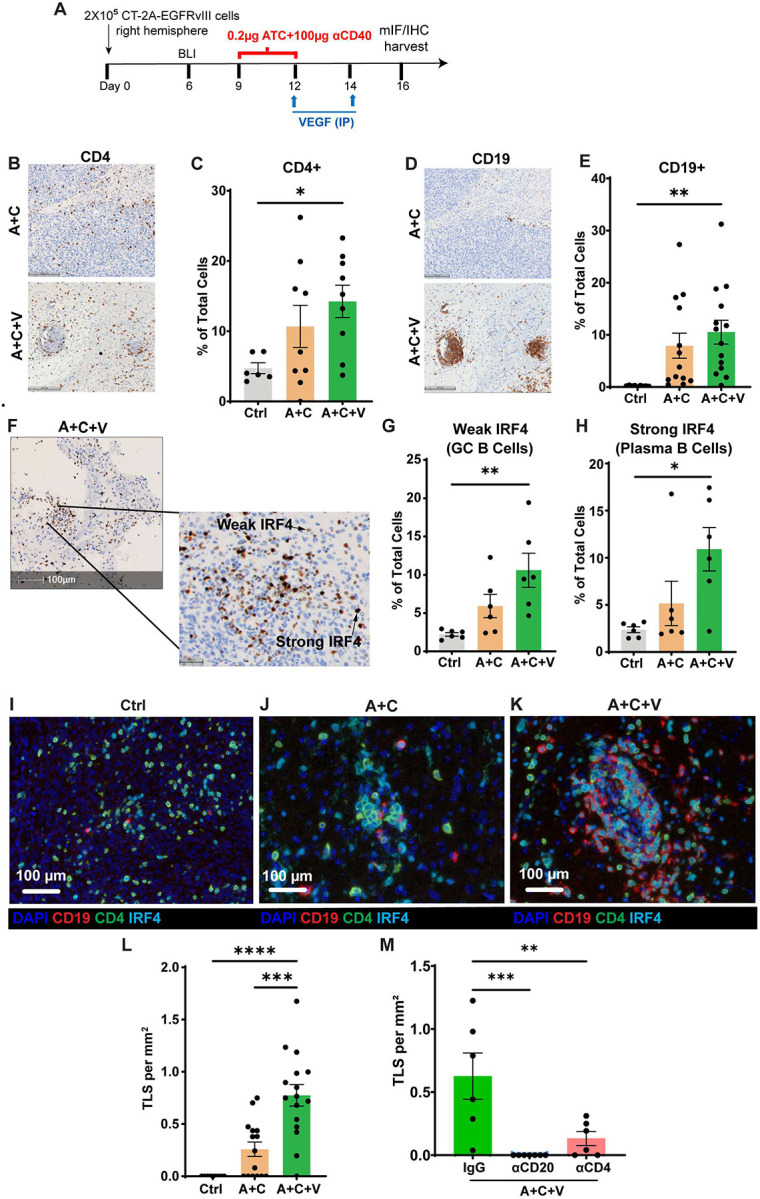
A+C+V therapy promotes the formation of TLS in the CT-2A-EGFRvIII tumor microenvironment. (**A**) Study outline for the treatment of CT-2A-EGFRvIII tumors in C57BL/6J female mice (n=4–5/group). (**B**-**C**) Representative IHC images of brain sections from CT-2A-EGFRvIII tumor-bearing mice treated with Ctrl, A+C, and A+C+V and stained with (**B**) CD4 antibody and (**C**) bar graph showing quantitative analysis of percentage of CD4^+^ cells. Scale bar, 200 μm. (**D**-**E**). Representative IHC images of brain sections from CT-2A-EGFRvIII tumor-bearing mice treated with Ctrl, A+C, and A+C+V and stained with (**D**) CD19 antibody and (**E**) bar graph showing quantitative analysis of percentage of CD19^+^ cells. Scale bar, 200 μm. (**F**-**H**) Representative IHC images of brain sections from CT-2A-EGFRvIII tumor-bearing mice treated with Ctrl, A+C, and A+C+V and stained with (**F**) IRF4 antibody and bar graph showing quantitative analysis of percentage of (**G**) weak IRF4^+^ cells as GC B cells and (**H**) strong IRF4^+^ cells as plasma B cells. Scale bar, 100 μm. (**I**-**K**) Representative mIF images of TLS in the tumor sections from CT-2A-EGFRvIII tumors treated with (**I**) Ctrl, (**J**) A+C, or (**K**) A+C+V, stained for DAPI (nucleus, blue), CD19 (red), CD4 (green), and IRF4 (blue). Scale bar, 100 μm. (**L**) Bar graph showing the frequency of TLS per mm^2^ in the CT-2A-EGFRvIII tumors treated with Ctrl, A+C, or A+C+V. (**M**) Bar graph showing the frequency of TLS per mm^2^ in the CT-2A-EGFRvIII tumors treated with A+C+V combined with IgG control or αCD20 (for B cell depletion) or αCD4 (CD4^+^ T cell depletion). Data represented as mean ± SEM. Data is from one experiment. Statistical analysis was performed using an unpaired t-test. **p<0.01, ***p<0.001.

Based on the TLS cellular gene signature observed in rGBM patients treated with bevacizumab plus irinotecan (Figure 1) and preclinical glioma treated with A+C+V (Figures 3 and 4), we next investigated whether A+C+V treatment could induce TLS formation in CT-2A-EGFRvIII glioma-bearing mice (Figure 5A). We first assessed A+C and A+C+V-induced changes in the lymphocyte compartment by IHC. Quantification of CD4^+^ T (*P* = 0.05) (Figures 5B and 5C) and CD19^+^ B (*P* = 0.01) (Figures 5D and 5E) cells in coronal sections of CT-2A-EGFRvIII tumor-bearing hemispheres from A+C and A+C+V groups revealed a significant increase in both cell types in the A+C+V group. Transient expression of the transcription factor IRF4 induces GCB cell differentiation, whereas sustained, higher concentrations of IRF4 promote PC generation^56^. Hence, we used IRF4 intensity to quantify GCB cell (weak IRF4) vs. PC (strong IRF4) generation post-A+C and A+C+V therapies (Figure 5F). IHC analysis demonstrated a significant increase in both GCB cells (*P* = 0.01) (Figures 5F and 5G and **Supplemental Figure 4A**) and PC (*P* = 0.059) (Figures 5F and 5H and **Supplemental Figure 4A**) in the A+C+V group than in the A+C group. To determine TLS formation, we next investigated the spatial organization of B cells and CD4^+^ T cells in the GBM TME following control, A+C, and A+C+V treatments (Figures 5I–5K). We noted TLS formation in both the A+C and A+C+V groups (Figures 5I–5K); however, the frequency of TLS per mm^2^ was significantly higher in the A+C+V group compared with the A+C group (*P* = 0.0003) (Figure 5L and **Supplemental Figures 4B-4D**). Further, in the A+C-treated brains, the TLS contained relatively fewer B cells (Figure 5J and **Supplemental Figure 4C**), whereas in the A+C+V group, the TLS were densely infiltrated with B cells, exhibited a distinct CD19^+^ B cell core, and were localized adjacent to CD4^+^ T cells, resembling early germinal center structures, which is a marker of mature TLS (mTLS)^57,58^ (Figure 5K and **Supplemental Figure 4D**). Notably, the mTLSs in the A+C+V group were predominantly located at the periphery of the tumor (**Supplemental Figure 4D**). Mature TLSs are known to support antitumor immunity and are associated with improved clinical outcomes when present at high density in the peritumoral region^57,59–61^.

Further, to determine whether treatment-induced B cells or CD4^+^ T cells are required for TLS formation, we depleted these cells with αCD20 or αCD4 antibodies in CT-2A-EGFRvIII tumor-bearing mice, followed by A+C+V treatment (**Supplemental Figure 5A**). Cellular depletion was confirmed by IHC (**Supplemental Figures 5B**-**5D**). Both B cell and CD4^+^ T cell depletion effectively abrogated TLS formation in A+C+V-treated mice (Figure 5M and **Supplemental Figures 5B-5D**), indicating that treatment-mediated recruitment of these cells is essential for TLS formation. Collectively, these findings demonstrate that αVEGF-mediated vascular remodeling promoted spatial reorganization, B and CD4^+^ T cell infiltration, and TLS formation following A+C+V therapy.

### A+C+V therapy generates an antitumorigenic myeloid TME

**Figure 6.**
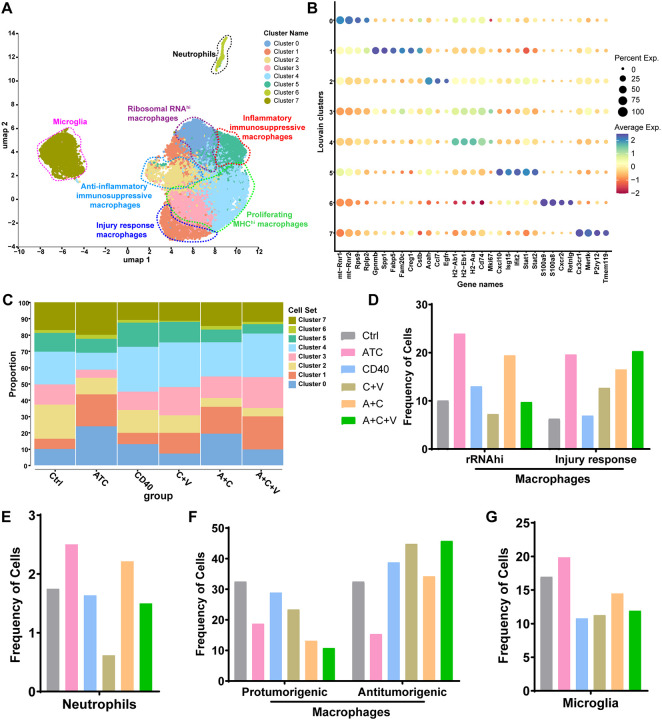
A+C+V therapy induces an antitumor phenotype in intratumoral myeloid subsets. (**A**) UMAP representation of different myeloid cell clusters in CT-2A-EGFRvIII tumors treated with Ctrl, ATC, αCD40, C+V, A+C, or A+C+V. A total of 20,434 myeloid cells across all treatment groups were subsetted from the scRNA-seq dataset (41,439 cells in total). (**B**) Dot plot showing average expression of cluster-specific marker genes in CT-2A-EGFRvIII tumors treated with Ctrl, ATC, αCD40, C+V, A+C, or A+C+V. (**C**) Bar graph showing the percentage of different myeloid cell clusters in CT-2A-EGFRvIII tumors post indicated treatments. (**D**-**G**) Bar graphs showing the frequency of (**D**) ribosomal RNA (rRNAhi) and injury response macrophages, (**E**) neutrophils, (**F**) pro-tumorigenic and anti-tumorigenic macrophages, and (**G**) microglia in CT-2A-EGFRvIII tumors post indicated treatments.

Myeloid cells are key mediators of immunosuppression in the GBM TME^62^, and both A+C^22^ and C+V^63^ therapies can reprogram myeloid subsets and improve antitumor response. Hence, we next investigated the influence of A+C+V therapy on the myeloid compartment in the CT-2A-EGFRvIII glioma model by subclustering 20,434 myeloid cells from the scRNA-seq dataset. UMAP analysis identified eight myeloid clusters based on conserved marker genes (Figures 6A and 6B); cluster 0 was ribosomal RNA high macrophages (*MT-RNR1, MT-RNR2, RPS9*, and *RPLP2*), cluster 1 was injury response macrophages (*GPNMB, SPP1, FABP5, FAM20C, CREG1*, and *CTSB*)^64^, cluster 2 was anti-inflammatory immunosuppressive macrophages (*AOAH, CCL7*, and *EGFR*)^65–67^, clusters 3 and 4, had overlapping markers, albeit with different expression levels, and consisted of proliferating MHCII-high macrophages (*H2-AB1, H2-EB1, H2-AA, CD74*, and *MKI67*)^68^, cluster 5 was immunosuppressive inflammatory macrophages (*CXCL10, ISG15, IFIT2, STAT1*, and *STAT2*)^69^, cluster 6 was neutrophils (*S100A9, S100A8, CXCR2* and *RETNLG*)^68^, and cluster 7 was microglia (*CX3CR1, MERTK, P2RY12*, and *TMEM119*)^70^ (Figure 6B). Ribosomal RNA macrophages (~19–24%) (Figures 6C [cluster 0] and 6D) and neutrophils (~2.2–2.5%) (Figures 6C [cluster 6] and 6E) were the highest in the ATC and A+C groups, indicating an antitumorigenic role for these myeloid subsets. Compared to the control group (~6%), the injury response macrophages were highest in all ATC groups (ATC, A+C, and A+C+V) (~16–20%) (Figures 6C [cluster 1] and 6D), suggesting that these macrophages contribute to tissue repair by clearing apoptotic cells and debris generated by ATC cytotoxicity^64^. In contrast, the protumorigenic immunosuppressive macrophages (clusters 2 and 5) were highest in the control group (~33%) and lowest in the A+C (~13%) and A+C+V (~11%) groups (Figures 6C and 6F). In line with antitumorigenic function, the antigen-presentation gene-enriched MHCII-high macrophages (clusters 3 and 4) were highest in the A+C+V group (~46%) (Figures 6C and 6F). Microglia (cluster 7) were highest in the ATC group (Figures 6C and 6G). Collectively, these findings confirm the functional plasticity of myeloid cells in the GBM TME and indicate that the antitumorigenic role of myeloid subsets is therapy-dependent. Finally, the antitumorigenic macrophages (cluster 1+3+4) were highest (~66%) while the protumorigenic macrophages (cluster 2+5) were lowest (~11%) in the A+C+V group (Figure 6C), indicating a role for A+C+V therapy in reprogramming the myeloid cells in the GBM TME.

### αVEGF enhances the antitumor efficacy of A+C therapy in gliomas

**Figure 7.**
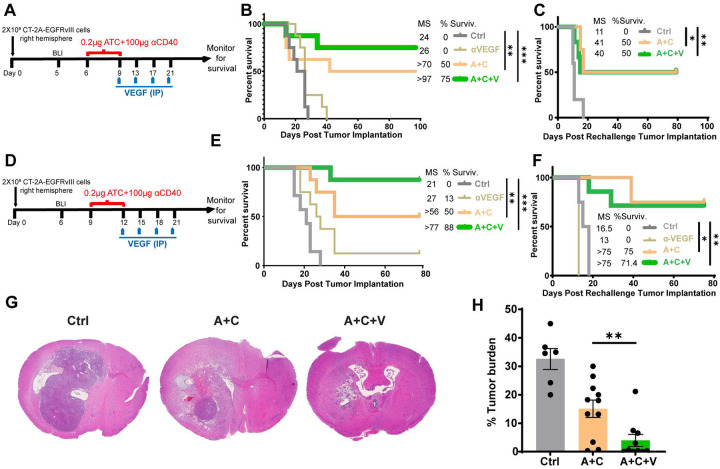
A+C+V therapy improves survival and generates a memory response in preclinical glioma models. (**A**) Study outline for day 6–9 CED treatment of CT-2A-EGFRvIII tumors in C57BL/6J female mice (small tumor model). (**B**) Kaplan-Meier curve depicting survival of CT-2A-EGFRvIII tumor-bearing mice (n=8/group), treated with Ctrl, αVEGF, A+C, or A+C+V. (**C**) Kaplan-Meier curve depicting survival of treated mice from (**B**) that had complete tumor regression for >75 days and were rechallenged with EGFRvIII-negative parental CT-2A tumor cells. Tumor-naïve mice (n=5) were used as controls. (**D**) Experimental outline for day 9–12 CED treatment of CT-2A-EGFRvIII tumors in C57BL/6J female mice (established tumor model). (**E**) Kaplan-Meier curve depicting survival of CT-2A-EGFRvIII tumor-bearing mice (n=7–8/group), treated with Ctrl, αVEGF, A+C, or A+C+V. (**F**) Kaplan-Meier curve depicting survival of treated mice from (**E**) that had complete tumor regression for >75 days and were rechallenged with parental CT-2A tumor cells. Tumor-naïve mice (n=5) were used as controls. (**G**) H&E-stained tumor sections of brains from day 9–12 CED-treated CT-2A-EGFRvIII tumors harvested 4 days post-CED treatment. (**H**) Percentage compositions of tumor cells of CT-2A-EGFRvIII tumor sections from respective treatment groups. *p<0.05, **p<0.01, ***p<0.001.

To determine whether the antitumorigenic immune milieu generated by A+C+V therapy translates into survival responses, orthotopic CT-2A-EGFRvIII glioma-bearing mice were treated post-tumor implantation on days 6–9 with control, αVEGF, A+C, and A+C+V as outlined (Figure 7A) and monitored for survival. Compared with control (median survival [MS] = 24 days) and αVEGF monotherapy (MS = 26 days), we observed increased survival with A+C therapy (MS > 70 days, *P* = 0.26 vs. control, *P* = 0.39 vs αVEGF), with 50% of the mice from the A+C group surviving long-term. αVEGF treatment improved A+C survival responses, with 75% of mice surviving long term with a significant increase in MS (MS > 97 days, *P* < 0.01 vs. control, *P* < 0.05 vs. αVEGF) (Figure 7B). To evaluate whether this translates into an adaptive memory response, surviving mice and naïve control mice were rechallenged on day 98 with CT-2A parental cells (lacking the ATC target antigen [EGFRvIII]) in the contralateral hemisphere. All naïve mice developed tumors and were euthanized, while 50% of A+C and A+C+V-treated mice survived and remained tumor-free until study termination (80 days post-rechallenge) (Figure 7C), confirming the generation of a memory response by A+C and A+C+V therapies.

To determine whether the A+C+V therapy can control the growth of established tumors, orthotopic CT-2A-EGFRvIII glioma-bearing mice were treated on post-tumor implantation days 9–12 with control, αVEGF, A+C, and A+C+V as outlined in Figure 7D. The control and αVEGF groups had a MS of 21 and 27 days, respectively, with 13% of mice surviving long term in the αVEGF group. However, the A+C group had 50% of the mice survive long term, with a MS of >56 days, and the A+C+V group further improved this, with 88% of mice surviving long term and a median survival of >77 days (Figure 7E). Tumor rechallenge studies in surviving mice and naïve control mice with tumor-antigen-negative CT-2A parental cells elicited a memory response, conferring tumor protection and long-term survival in 75% and 71% of mice treated with A+C and A+C+V, respectively, but not in the αVEGF group (Figure 7F). Tumor control resulting in improved survival in the A+C+V group was next assessed by H&E staining. Brains from CT2A-EGFRvIII-bearing mice treated with A+C and A+C+V were harvested 4 days post-therapy (day 16), and coronal sections were histologically examined for tumor burden. Compared to A+C-treated mice (40% tumor burden), A+C+V-treated mice exhibited a significantly reduced tumor burden (10%) (Figures 7G and 7H), confirming the robust antitumor response of A+C+V against established gliomas.

The antitumor efficacy of A+C+V therapy was evaluated in a second established glioma model, GL261-EGFRvIII^22^, following the study outline in **Supplemental Figure 6A**. The control and A+C groups had median survival of 19 and 31 days, respectively, with one long-term survivor (17%) in the A+C group (**Supplemental Figure 6B**). In contrast, the A+C+V group had an MS >75 days (*P* < 0.01), with 67% of mice surviving long-term (**Supplemental Figure 6B**). Tumor rechallenge studies in surviving mice with tumor-antigen-negative GL261 parental cells showed long-term survival in 100% of mice from A+C (n=1) and A+C+V (n=4) groups, indicating the generation of a durable antitumor memory response (**Supplemental Figure 6C**). In contrast, all control naïve mice implanted with GL261 parental cells succumbed to the tumor (MS = 14 days) (**Supplemental Figure 6C**). Together, these findings demonstrate that αVEGF-mediated vascular remodeling enhances A+C antitumor immune responses and significantly improves survival in glioma models.

## Discussion

GBM remains one of the most lethal solid tumors, largely due to its profoundly immunosuppressive microenvironment and aberrant vasculature, which together restrict effective immune surveillance and therapeutic responsiveness^2^. Although anti-angiogenic therapy with bevacizumab is widely used in rGBM, its immunological consequences have remained poorly defined. Our findings show that VEGF blockade, in combination with chemotherapy or cytotoxic immunotherapy (A+C), is a critical modulator of the vascular-immune axis in GBM, linking vascular remodeling to immune activation and TLS formation, underscoring the importance of A+C+V therapy for improving survival in patients with GBM.

VEGF blockade through Bevacizumab has been used in GBM patients to treat edema since 2009^71^, but herein we present a model in which αVEGF enhances cytotoxic or immunotherapy-induced TLS formation and antitumor responses. While our patient data demonstrate that VEGF blockade, together with chemotherapy, is associated with TLS-linked immune remodeling, our preclinical studies focused on VEGF inhibition in combination with cytotoxic and immune-activating therapies. Thus, we hypothesize that VEGF blockade alone is insufficient to induce TLS formation in GBM, as bevacizumab alone does not result in improved overall survival^72^. Instead, our data support a model in which VEGF inhibition acts as an immunological amplifier, normalizing the vasculature and enhancing lymphatic endothelial function, thereby enabling spatial immune organization initiated by cytotoxic immune-stimulatory therapies.

VEGF-driven angiogenesis causes endothelial cell instability, basement membrane disruption, and inadequate pericyte coverage, resulting in mechanistically dysfunctional GBM tumor vasculature^73^. This aberrant vascular architecture leads to impaired perfusion, elevated interstitial pressure, and heterogeneous hypoxia, thereby limiting effective delivery of oxygen and therapeutics and promoting therapy resistance^74^. VEGF inhibition has the potential to restore endothelial integrity and pericyte support, transiently normalizing vascular function and improving tissue oxygenation^75^; however, excessive angiogenic suppression can instead induce vessel regression, reducing perfusion and counteracting therapeutic benefit^76^. These opposing mechanisms highlight the importance of defining optimal therapeutic combinations and timing to exploit vascular normalization while preserving functional perfusion.

Transcriptomic analysis of rGBM patients showed that clinical response to bevacizumab plus irinotecan is associated with a pronounced shift toward an antitumor immune landscape. Responders exhibited increased infiltration of effector cell states, such as plasma cells, Tfh and CD8^+^ T cells, and M1 macrophages, accompanied by a reduction in pro-tumorgenic cells such as naïve and regulatory T cells, and M2 macrophages. Notably, Tfh and plasma cells, central drivers of TLS formation, were among the most significantly enriched populations, suggesting that VEGF blockade and chemotherapy combination in rGBM patients promotes an organized antitumorigenic immune milieu in the GBM microenvironment. Importantly, pooling responders and non-responders obscured these immune distinctions, underscoring the need for outcome-based immune stratification when evaluating the immunomodulatory effects of anti-angiogenic therapies in GBM.

Mechanistic studies in orthotopic glioma models demonstrated that adding VEGF blockade to intratumoral A+C therapy profoundly remodeled the tumor vasculature. A+C+V treatment increased pericyte coverage and reduced endothelial proliferation, consistent with vascular normalization rather than vessel depletion^35^. Such stabilization of the vascular network is increasingly recognized as essential for immune cell trafficking and increasing the effectiveness of immunotherapy^33^. In parallel, single-cell transcriptomic analyses revealed enrichment of endothelial subsets expressing *CD34* and *VEGFC*, molecules associated with lymphatic maturation, antigen drainage, and T-cell priming in deep cervical lymph nodes^43^. Additionally an endothelial cell cluster characterized by upregulation of immune-activated genes, including *IL1B*, *SELE*, and *SELP*, and associated with systemic inflammation, has been identified in the tumor cores of patients with GBM^77^. Similarly, upregulation of adhesion molecules (*SELP* and *SELE*) and chemokines (*CXCL12*), which are involved in leukocyte transmigration, further suggests that VEGF blockade augments A+C therapy-induced immune infiltration by actively remodeling the vascular interface^44,78^.

Vascular reprogramming was accompanied by a pronounced polarization of the adaptive immune response toward a Tfh-germinal center axis. A+C+V therapy selectively expanded memory-like CD4^+^ Tfh cells, which are known to orchestrate B-cell recruitment, germinal center formation, and affinity maturation within TLS^25,50^. Concurrently, the B-cell compartment shifted from progenitor populations toward germinal center B cells, plasmablasts, and plasma cells, with elevated RAG1/2 expression indicative of active immunoglobulin gene rearrangement^54,55^. While αCD40 signaling alone was sufficient to initiate B-cell activation and differentiation, VEGF blockade appeared to provide the spatial and vascular context necessary for sustained germinal center organization and functional TLS maturation. Consistent with this model, histological analyses revealed that A+C therapy induced immature TLS formation, characterized by disorganized aggregates of lymphocytes^79^, whereas the addition of VEGF blockade significantly increased TLS density and structural organization, resulting in mature, germinal center-like TLS. Mature TLS were preferentially localized to the tumor periphery, a region increasingly recognized as a critical immunological niche associated with improved clinical outcomes across multiple cancers^61^. In untreated primary GBM patient samples, TLS can be found mostly associated with the meninges^80–83^, and an increase in mature TLS results in improved overall survival in GBM patients^80,82^.

In GBM, B cells are known to exhibit a regulatory phenotype^84^, and high B cell receptor sequence diversity has been associated with reduced survival^85^. Conversely, in preclinical GBM models, LIGHT/TNFSF14 therapy promoted TLS formation with an increased T cell-to-B cell ratio, leading to effective anti-tumor immunity^86^. Prior studies have shown that αCD40 treatment induces TLS formation both clinically^87^ and preclinically^81^; however, the preclinical studies demonstrated an increase in CD11b^+^ suppressive B cells, and failed to improve overall survival in two orthotopic GBM mouse models^81^. This suggests that αCD40 alone is insufficient to elicit robust antitumor responses in GBM. Here, we show that A+C+V-induced TLS formation is accompanied by an increase in CD4^+^ Tfh cells compared with all other treatment groups. This regimen may therefore enhance B cell diversity and antibody production, potentially shifting B cells from a pro-tumoral to an anti-tumoral state and improving survival compared with αCD40 monotherapy. To test this, we measured tumor-specific IgG levels in plasma 4 days post-A+C+V therapy, but did not detect antitumor IgG. Given that peripheral germinal center responses typically require 10–14 days to generate high-affinity antibodies^88,89^, it is likely that the short interval between A+C+V therapy and analysis was insufficient to detect IgG production. Since low-dose bevacizumab (αhuman-VEGF) is included in our GBM immunotherapy trials to control symptomatic persistent peritumoral inflammation, plasma antitumor IgG levels and Tfh-plasma cell signatures in peripheral blood mononuclear cells will be monitored as markers of TLS formation and antitumor response, both pre- and post-bevacizumab, in patients enrolled in our ongoing ATC + αCD40 (clone 2141-V11) clinical trials in newly diagnosed (NCT05734560) and recurrent (NCT04547777 and NCT06455605) IDHwt GBM patients.

Beyond lymphoid remodeling, A+C+V therapy also reprogrammed the myeloid compartment toward an antitumorigenic state. We previously demonstrated that αCD40 promotes a proinflammatory phenotype in tumor-associated macrophages and elicits tumor-specific CD8^+^ T cell immunity in the GBM microenvironment^22^. Herein, we expanded on those findings, as A+C+V combination therapy reduced immunosuppressive macrophage subsets while enriching antigen-presenting, MHCII^high^ macrophages and injury-response macrophages associated with debris clearance and tissue repair. Notably, these changes reflected functional reprogramming rather than alterations in myeloid cell abundance, highlighting the plasticity of the GBM myeloid compartment and its capacity to support adaptive immune responses when appropriately conditioned. Such myeloid rebalancing likely reinforces TLS function by enhancing antigen availability and alleviating local immunosuppression within the TME^64,69^. The immune remodeling induced by A+C+V therapy translated into robust therapeutic benefit, with significantly improved survival, reduced tumor burden, and the generation of durable immunological memory across different glioma models. Importantly, long-term survivors rejected antigen-negative tumor rechallenge, indicating effective antigen spreading and the establishment of systemic immune memory—outcomes that remain exceedingly rare in GBM. These findings underscore the functional relevance of TLS-driven immunity and vascular normalization in sustaining long-term tumor control in this otherwise treatment-resistant disease.

Additionally, our scRNA-seq analysis identified a proinflammatory macrophage subset (expressing *CXCL10*, *ISG15*, and *IFIT2*) driven by aberrant *IFNG* signaling via *STAT1* (Figures 6B and 6C). The highest frequency of these proinflammatory macrophages was observed in the αCD40 and C+V treatment groups, suggesting that the proinflammatory milieu induced by CD40 costimulation, when used as a single agent in aggressive tumors such as GBM, may be counterproductive—potentially promoting tumor progression, immune evasion, and reduced patient survival.

In summary, our study demonstrates that VEGF blockade not only remodels tumor vasculature but also fundamentally redefines the immune architecture of gliomas by promoting TLS formation and enhancing adaptive immunity. Through vascular normalization and the establishment of structured immune niches, αVEGF synergizes with ATC and αCD40 to achieve potent and durable antitumor responses. Our work highlights a promising translational avenue for combining vascular-targeted, cytotoxic, and immunomodulatory therapies to overcome immune resistance and improve outcomes in GBM.

## Material and Methods

### Cell line and culture conditions

Mouse brain tumor cell lines^22^ CT-2A, GL261, CT-2A-EGFRvIII, and GL261-EGFRvIII were passaged at confluence with Accutase Cell Detachment Solution (BD Bioscience). The cell lines were cultured in DMEM-high glucose medium (Thermo Fisher Scientific) supplemented with 10% FBS (Thermo Fisher Scientific) in an incubator maintained at 37 °C with 5% carbon dioxide. The cell lines were tested for rodent pathogens and authenticated by whole-exome sequencing^90^. The cell culture supernatants were tested for mycoplasma infection. After thawing, all cell lines were maintained in culture for <10 passages.

### Preparation of recombinant ATC

ATC was expressed in *Escherichia coli* BL21 (λ DE3) (Agilent Technologies) under the control of a T7 promoter. D2C7-IT from inclusion bodies was reduced, refolded, and further purified as monomers by ion exchange and size exclusion chromatography, as described^91^. Last, endotoxin removal from the purified D2C7-IT was achieved with ActiClean Etox resin (Sterogene). The purity of the final D2C7-IT preparation was >95% as determined by SDS–polyacrylamide gel electrophoresis and high-performance size exclusion chromatography.

### Mouse serum albumin

The recombinant mouse serum albumin (MSA) protein (Albumin Bioscience) was solubilized in sterile 1x phosphate-buffered saline (PBS) (Thermo Fisher Scientific), filter-sterilized, vialed, and stored at 4°C.

### Mice

All experiments were done in accordance with the Institutional Animal Care and Use Committee of Duke University Medical Center (A019-24-01). Animals were maintained in a barrier facility under pathogen-free conditions in accordance with NIH guidelines. Female C57BL/6J mice were purchased from The Jackson Laboratory. All mice used were 7–8 weeks old and weighed 16–20 gm at the start of the study.

### Intracranial tumor implantation

For tumor implantation, mice were anesthetized and secured onto a digital mouse stereotactic frame. A 0.5-inch midline incision was made to expose the skull. A cordless drill (Dremel, no. 21026859) equipped with a carbide bur operative friction grip 1/4 (Henry Schein, no. 1007205) was used to create a pilot hole 0.5 mm anterior and 2.0 mm lateral to the bregma. A 25 μL Hamilton syringe (Hamilton Company Model 710 RN SYR, no.7638–01) with a 27-gauge needle, mounted on an automatic Quintessential Stereotaxic Injector, was vertically inserted into the pilot hole and lowered to a depth of 3.3 mm below the skull. A total of 2×10^5^ CT-2A-EGFRvIII or 1×10^5^ GL261-EGFRvIII mouse glioma cells were injected in 5 μl of 1x PBS containing 3% methylcellulose (Sigma, no. 9004-67-5) at a rate of 3.33 μl per min. The drill hole was sealed with Medical Bone wax (Medline no. DYNJBW26), and the incision site was closed using veterinary surgical adhesive (Covertus, no.031477).

### Bioluminescence imaging (BLI) and randomization

For the A+C+V therapy studies, 5 or 6 days post-tumor implantation, mice were anesthetized, injected with 3 mg D-Luciferin, Potassium Salt (GoldBio) dissolved in 100 μl of PBS, and imaged for tumor presence using a Perkin Elmer IVIS Lumina III In Vivo Imaging System. Mice were then randomized into different treatment groups (N=6–8 per group for antitumor efficacy studies and 3–8/group for immunohistochemistry and RNA-Seq studies) by total flux using an open-access randomization software (Urbaniak, G.C., & Pious, S. (2013). Research Randomizer (Version 4.0) [Computer software], randomizer.org).

### Convection-enhanced delivery (CED) treatment

On day 6 (CED days 6–9) or day 9 (CED days 9–12) post-tumor implantation, mice were treated with ATC (0.2 μg) and αCD40 (100 μg; clone FGK4.5/FGK45, Bio × Cell) combination therapies by CED using Alzet Micro-Osmotic Pumps (model 1007D, Durect Corporation) at a rate of 0.5 μl/h for 72 h. Control groups were treated with 2% mouse serum albumin (MSA)–PBS with 100 μg of rat IgG2a antibody (Bio × Cell, InVivoPlus clone 2A3). At the end of 72-h CED, the Alzet Micro-Osmotic Pumps were removed, and animals were treated with 2.5 mg/kg of body weight of αVEGF antibody (B20–4.1.1, a monoclonal antibody cross-reactive with human and murine VEGF provided by Genentech) or isotype control delivered by intraperitoneal injection every 3 days for 1 to 4 doses. Mice were monitored long-term (75–80 days) for survival.

### Antitumor efficacy determination

The treatment-derived antitumor response to intracranial tumors was assessed by the percentage increase in survival time to weight loss or a specific neurologic endpoint (i.e., seizure activity, repetitive circling, or other subtle changes such as a decrease in appetite). For survival studies, mice were monitored every 48 hr post tumor implantation, increasing to twice daily with the onset of neurologic symptoms and/or weight loss exceeding 15% of initial body mass, at which time, the mice were euthanized.

### Tumor rechallenge studies

Mice that survived symptom-free for >70 days after initial tumor implantation were rechallenged on days 75 to 80 and were monitored for survival with age-matched naïve control mice (N=4). 1 × 10^5^ antigen-negative CT-2A (denoted as CT-2A parental cells) or 7.5 × 10^4^ antigen-negative GL261 (denoted as GL261 parental cells) cells were injected at the same coordinates in the contralateral hemisphere. Mice were monitored long-term for survival.

### Depletion of B cells or CD4 T cells

Mice received 12.5mg/kg anti-mouse CD20 mAb (clone MB20–11, Bio × Cell, cat# BE0356) or isotype control rat IgG2c mAb (clone DV5–1, Bio × Cell, cat# BE0366) by intraperitoneal injection on days 5 and 12 relative to tumor implantation. For CD4 depletion, 12.5mg/kg of anti-mouse CD4 (clone GK1.5, Bio × Cell, cat# BE0003–1) was administered i.p. on days 5, 9, 12, and 14 relative to tumor implantation. Depletion efficiency was confirmed by immunohistochemistry using anti-CD19 (clone 1D3, BD Biosciences, cat# 553783) and anti-CD4 (clone RM4–5, BD Biosciences, cat# 553647) staining of brain sections.

### Tissue Preparation

For immunohistochemistry and multiplex immunofluorescence tissue preparation, 3–5 mice/group underwent the same tumor implantation and indicated therapy-administration protocols. Mice were euthanized on post-tumor implantation day 16 (post-CED therapy day 4) as required per each study. Orthotopic tumor-bearing mice were euthanized with isoflurane inhalation and subjected to cardiac perfusion using 1x PBS. The brains were then removed for downstream analysis.

### Immunohistochemistry (IHC)

For IHC analysis, whole brains were fixed in 10% neutral buffered formalin (NBF) (VWR) for 24-to 48-hr, rinsed with 1x PBS, and transferred to 70% ethanol (EtOH) for 72 hr. Brains were then bisected coronally at the site of tumor implantation and submitted for paraffin embedding to the Duke Pathology Core Laboratory. The formalin-fixed paraffin-embedded (FFPE) blocks were then submitted to the Preston Robert Tisch Brain Tumor Center Biorepository for serial sectioning (5 μm thickness), and one slide was chosen for Hematoxylin and Eosin (H&E) staining to identify and characterize tumor infiltration. The percentage of tumor burden in selected H&E slides was quantified through the HALO v3.5 (Indica Labs) annotation tool area measurement. Immunohistochemistry analysis was performed via standard methods described below. Representative unstained serial FFPE sections were stained with CD31 Ab (clone D8V9E, Cell Signaling Technology, 1:400), SMA Ab (clone D4K9N, Cell Signaling Technology, 1:200), CD19 Ab (clone D4V4B, Cell Signaling Technology, 1:400), CD4 Ab (clone D7D2Z, Cell Signaling Technology, 1:100), IRF4 Ab (clone E8H3S, Cell Signaling Technology, 1:100), and CD8 Ab (clone D4W2Z, Cell Signaling Technology, 1:400) using automated IHC techniques on a BOND^™^ RXm Processing Module (Leica Microsystems), utilizing the Bond^™^ Polymer Refine Detection kit (Leica Microsystems). Bond Epitope Retrieval Solution 2 (ER2) (EDTA buffer, pH 9.0; Leica Microsystems) was used for CD31 Ab, SMA Ab, CD4 Ab, and IRF4 Ab. Bond Epitope Retrieval Solution 1 (ER1) (citrate-based buffer, pH 6.0; Leica Microsystems) was used for CD19 Ab and CD8 Ab. The IHC slides were scanned using the Vectra 3.0 System (Akoya Biosciences), and the qptiff files were viewed and saved in Phenochart 1.1 whole slide viewer (Akoya Biosciences). Image analysis and cell quantification were performed using HALO v3.5 (Indica Labs). Area measurements of SMA were performed using the Area Quantification FL v2.3.3 module. The percentage of marker-positive area of the tumor hemisphere was used for quantification. CD31 vessel length was quantified using the Object Colocalization v2.1.5 module. All module parameters, including nuclear size, cytoplasmic expansion radius, channel thresholds, and classification settings, were kept constant for all samples to ensure comparability.

### Multiplex immunofluorescence staining

Representative unstained serial FFPE sections (5 μm thickness) were stained using automated IHC techniques on BOND^™^ RXm Processing Module, utilizing the Bond Research Detection kit (Leica Microsystems) and Opal fluorophores (Akoya Biosciences). In brief, FFPE sections were deparaffinized, hydrated with alcohol, and subjected to heat-induced epitope retrieval with Bond ER1 (for CD19 Ab) or ER2 (for SMA Ab, CD31 Ab, IRF4 Ab, and CD4 Ab). Slides were then washed with Bond wash solution, and non-specific protein binding was blocked with protein block (5 min). For SMA and CD31 co-staining, the sections were sequentially incubated with SMA Ab (clone D4K9N, Cell Signaling Technology, 1:1000, 30 min), Rabbit Polymer (10 min), Opal 620 (10 min), and CD31 Ab (clone D8V9E, Cell Signaling Technology, 1:4000, 30 min), Rabbit Polymer (10 min), Opal 690 (10 min) Ab fluorophore combination. For IRF4, CD4, and CD19 co-staining, the sections were sequentially incubated with IRF4 Ab (clone E8H3S, Cell Signaling Technology, 1:500, 30 min), Rabbit Polymer (10 min), Opal 520 (10 min), CD4 Ab (clone D7D2Z, Cell Signaling Technology, 1:250, 30 min), Rabbit Polymer (10 min), Opal 620 (10 min), and CD19 Ab (clone D4V4B, Cell Signaling Technology, 1:2000), Rabbit Polymer (10 min), Opal 690 (10 min) Ab fluorophore combination. The nuclei were subsequently stained with spectral DAPI (4,6-diamidino-2-phenylindole) solution (Akoya Biosciences). The sections were cover-slipped using Vectashield HardSet Antifade mounting media (Vector Laboratories). The slides were scanned using the Vectra 3.0 System, and image analysis was performed with Inform (Akoya) and HALO v3.5 (Indica Labs). Cell classification and phenotype detection were conducted using the Highplex FL v4.2.3 module. For each slide, nuclear seed points were identified using the DAPI channel, and cytoplasmic expressions were optimized for each fluorophore to ensure accurate phenotype calling. Intensity thresholds for all channels were established using matched control tissues and applied uniformly across the dataset.

TLS numbers were quantified by IRF4, CD4, and CD19 co-staining and defined as clusters of CD4^+^ T cells and CD19^+^ B cells with diameters greater than 150 μm. IRF4 intensity above the 50^th^ percentile was classified as strong staining, indicative of a plasma cell (PC)–like phenotype, whereas IRF4 intensity below the 50^th^ percentile was classified as weak staining, consistent with a germinal center B-cell (GCB) phenotype.

### Single-cell RNA-sequencing (sc-RNAseq)

For single-cell RNA-sequencing analysis, 3–4 mice/group underwent tumor implantation and treatment with Control, ATC, αCD40, ATC+αCD40, αCD40+αVEGF, or ATC+αCD40+αVEGF. Treated mice were euthanized on post-treatment day 4, tumor-bearing hemisphere was isolated and dissociated as previously described^22^. The final cell pellet was washed with 1x PBS, counted, adjusted to 0.1–1×10^6^ live cells/sample, and fixed with Evercode Cell Fixation v3 kit (Parse Biosciences). Post-fixation, the samples were processed using split-pool combinatorial barcoding following the manufacturer’s protocol (Evercode Whole Transcriptome V3 kit, Parse). The scRNA-seq gene expression libraries were sequenced (Illumina NovaSeq × Plus; University of Colorado Cancer Center, Genomics & Microarray Core Facility) to an average depth of ~50,000 reads/cell with a 150 bp paired-end read configuration. The raw sequencing data were demultiplexed, processed, and mapped to the GRCm39 reference using the Parse Trailmaker pipeline. Further quality control, clustering, and visualizations were performed using Trailmaker. Following cluster annotation using canonical markers, the myeloid, T cell, B cell, NK cell, endothelial, and tumor clusters were subsetted and analyzed using Trailmaker.

### Public Dataset Analysis

Transcriptomic data from glioblastoma (GBM) patients were obtained from the Gene Expression Omnibus (GEO; accession number GSE79671)^92^. Treatment response was evaluated in the original study according to the Response Assessment in Neuro-Oncology criteria, with confirmation on a subsequent MRI. Responders were defined as patients with complete or partial response, and non-responders as patients with stable disease or progressive disease. To estimate the proportions of tumor-infiltrating immune cell types, the TPM-normalized counts were determined and used as input into the CIBERSORT^93^ and TIMER^94^ deconvolution programs, using the human GBM cancer type as a reference dataset. Group differences were visualized as box plots generated in R (version 4.5.1), and statistical significance was assessed using two-tailed *t*-tests.

### Statistical Analysis

GraphPad PRISM software was used for data visualization and statistical analysis. Pairwise comparisons of survival curves were performed using the generalized Wilcoxon test. Data were compared between groups using the two-way analysis of variance (ANOVA) or unpaired t-test. Data indicate mean values ± standard error of the mean (SEM). The number of animals in each group and P values are reported in the Figure legends, and no outliers or other data points were excluded.

### Materials availability

Mouse tumor cell lines generated in this study will be made available to the broader scientific community upon request to the corresponding author with a completed materials transfer agreement.

## Supplementary Material

Supplementary Files

This is a list of supplementary files associated with this preprint. Click to download.

- SupplementaryFigure1Final.tif
- SupplementaryFigure2Final.tif
- SupplementaryFigure3Final.tif
- SupplementaryFigure4Final.tif
- SupplementaryFigure5Final.tif
- SupplementaryFigure6Final.tif

**Supplemental Figure 1. Lack of immune activation in pre- vs. post-treatment tumors from all rGBM patients treated with bevacizumab and chemotherapy combination.** Box plots showing the estimated cell-type proportions from TIMER3 deconvolution of pre-treatment (n=20) and post-treatment (n=16) tumor transcriptomic data from rGBM patients (n=20) treated with a combination of bevacizumab and chemotherapy.

**(A)** Box plots of plasma B cells, Tfh cells, CD8^+^ T cells, γδ T cells, and M1 macrophages.

**(B)** Box plots of naïve CD4^+^ T cells, regulatory T cells, and Th17 cells.

**(C)** Box plots of M2 macrophages, endothelial cells, and CAFs.

Box plots display the median (center line) with the interquartile range (IQR). Statistical analysis was performed using an unpaired t-test **p<0.01.

**Supplemental Figure 2. A+C+V therapy enhances pericyte coverage and promotes vascular normalization.** (**A-C, Left**) Representative mIF images of tumor-bearing hemispheres from CT-2A-EGFRvIII tumor-bearing mice treated with (**A**) Ctrl, (**B**) A+C, or (**C**) A+C+V and stained with DAPI, SMA, and CD31 antibodies. Scale bar, 200 μm.

(**A-C, Right**) Magnified images of two different areas within the tumor-bearing hemisphere from (**A**) Ctrl, (**B**) A+C, or (**C**) A+C+V. Within each treatment group the top panel shows DAPI, SMA, and CD31 staining, middle panel shows SMA staining, and bottom panel shows CD31 staining. Scale bar, 100 μm.

(**D-E**) Representative IHC images of brain sections from CT-2A-EGFRvIII tumor-bearing mice treated with **(D)** A+C or (**E**) A+C+V and stained with CD31 (top) or SMA (bottom) antibodies. Scale bar, 100 μm.

**Supplemental Figure 3. A+C+V therapy reshapes the GBM tumor microenvironment and enhances adaptive immune infiltration.** **(A)** UMAP visualization of single-cell RNA sequencing data from CT-2A-EGFRvIII tumors treated with Ctrl, ATC, αCD40, C+V, A+C, or A+C+V, demonstrating six major cell populations.

**(B)** Dot plot showing the expression of canonical marker genes used to define tumor (*EGFR*), endothelial (*CD34*), myeloid (*AIF1*, *CX3CR1*, *TMEM119*), NK (*NKG7*), B (*CD19*, *CD22*), and T (*CD4*, *CD8A*, *CD8B*) cell clusters.

**(C)** Relative cell composition in individual clusters across treatment groups.

**(D)** Proportion of B and T cells in CT-2A-EGFRvIII tumors treated with Ctrl, A+C, or A+C+V.

**(E)** Proportion of tumor and NK cells in CT-2A-EGFRvIII tumors treated with Ctrl, A+C, or A+C+V.

**(F)** Proportion of endothelial and myeloid cells in CT-2A-EGFRvIII tumors treated with Ctrl, A+C, or A+C+V.

Data are derived from 41,439 high-quality single cells pooled from 3–4 mice per group.

**Supplemental Figure 4. A+C+V therapy increases TLS density and B cell differentiation into germinal center B cells and plasma cells. (A)**. Representative IHC images of brain sections from CT-2A-EGFRvIII tumor-bearing mice treated with Ctrl, A+C, and A+C+V and stained with IRF4 antibody. Weak IRF4^+^ cells are GC B cells, and strong IRF4+ cells are plasma B cells. Scale bar, 100 μm.

(**B-D, Left**) Representative mIF images of tumor-bearing hemispheres from CT-2A-EGFRvIII tumor-bearing mice treated with (**B**) Ctrl, (**C**) A+C, or (**D**) A+C+V and stained with DAPI, CD19, CD4, and IRF4 antibodies. TLSs are highlighted in yellow boxes. Scale bar, 1000 μm.

(**B-D, Right**) Magnified images of two different areas within the tumor-bearing hemisphere from (**B**) Ctrl, (**C**) A+C, or (**D**) A+C+V. Within each treatment group, the panel shows from top to bottom, all markers, CD19, CD4, and IRF4 staining. Scale bar, 100 μm.

**Supplemental Figure 5. B cell and CD4**^**+**^ **T cell depletion abrogates A+C+V–induced TLS formation** (**A**) Study outline for the treatment of CT-2A-EGFRvIII tumors in C57BL/6J female mice (n=3–4/group).

(**B**-**C**) Representative IHC images of brain sections from CT-2A-EGFRvIII tumor-bearing mice treated with (B) A+C+V+αIgG, (C) A+C+V+αCD20, or (D) A+C+V+αCD4. Scale bar, 500 μm

**Supplemental Figure 6. A+C+V therapy improves survival and mediates robust tumor control in the GL261 preclinical glioma model.** (**A**) Study outline for the treatment of GL261-EGFRvIII tumors in C57BL/6J female mice (n=6/group).

(**B**) Kaplan-Meier curve depicting survival of GL261-EGFRvIII tumor-bearing C57BL/6J mice (n=6/group), treated with Control, A+C, or A+C+V.

(**C**) Kaplan-Meier curve depicting survival of treated mice from (**B**) that achieved tumor regression for >75 days, followed by rechallenge with the CT-2A tumor cells. Tumor-naïve mice (n=5) were used as controls. *p<0.05, **p<0.01.

## Data Availability

The datasets generated and/or analyzed in the current study will be deposited in GEO (GSE327261) upon publication. This paper does not report original code. Any additional information required to reanalyze the data reported in this paper is available from the corresponding author upon request.

## References

[1] Price M., CBTRUS Statistical Report: Primary Brain and Other Central Nervous System Tumors Diagnosed in the United States in 2017–2021. Neuro Oncol 26, vi1–vi85 (2024). 39371035 10.1093/neuonc/noae145 PMC11456825

[2] Chuntova P., Unique challenges for glioblastoma immunotherapy-discussions across neuro-oncology and non-neuro-oncology experts in cancer immunology. Meeting Report from the 2019 SNO Immuno-Oncology Think Tank. Neuro Oncol 23, 356–375 (2021). 33367885 10.1093/neuonc/noaa277 PMC7992879

[3] Ghosh M., Lenkiewicz A.M. & Kaminska B. The Interplay of Tumor Vessels and Immune Cells Affects Immunotherapy of Glioblastoma. Biomedicines 10(2022). 10.3390/biomedicines10092292 PMC9496044 36140392

[4] Onishi M., Ichikawa T., Kurozumi K. & Date I. Angiogenesis and invasion in glioma. Brain Tumor Pathol 28, 13–24 (2011). 21221826 10.1007/s10014-010-0007-z

[5] Das S. & Marsden P.A. Angiogenesis in glioblastoma. N Engl J Med 369, 1561–1563 (2013). 24131182 10.1056/NEJMcibr1309402 PMC5378489

[6] Kaur B., Hypoxia and the hypoxia-inducible-factor pathway in glioma growth and angiogenesis. Neuro Oncol 7, 134–153 (2005). 15831232 10.1215/S1152851704001115 PMC1871894

[7] Ghalehbandi S., Yuzugulen J., Pranjol M.Z.I. & Pourgholami M.H. The role of VEGF in cancer-induced angiogenesis and research progress of drugs targeting VEGF. Eur J Pharmacol 949, 175586 (2023). 36906141 10.1016/j.ejphar.2023.175586

[8] Ohm J.E. & Carbone D.P. VEGF as a mediator of tumor-associated immunodeficiency. Immunol Res 23, 263–272 (2001). 11444391 10.1385/IR:23:2-3:263

[9] Mahaki H., Targeting VEGF signaling for tumor microenvironment remodeling and metastasis inhibition: Therapeutic strategies and insights. Biomed Pharmacother 186, 118023 (2025). 40164047 10.1016/j.biopha.2025.118023

[10] Meadows K.L. & Hurwitz H.I. Anti-VEGF therapies in the clinic. Cold Spring Harb Perspect Med 2(2012). 10.1101/cshperspect.a006577 PMC3475399 23028128

[11] Taal W., Single-agent bevacizumab or lomustine versus a combination of bevacizumab plus lomustine in patients with recurrent glioblastoma (BELOB trial): a randomised controlled phase 2 trial. Lancet Oncol 15, 943–953 (2014). 25035291 10.1016/S1470-2045(14)70314-6

[12] Sepúlveda J.M., A phase II study of feasibility and toxicity of bevacizumab in combination with temozolomide in patients with recurrent glioblastoma. Clin Transl Oncol 17, 743–750 (2015). 26033428 10.1007/s12094-015-1304-0

[13] Huang Y., Vascular normalizing doses of antiangiogenic treatment reprogram the immunosuppressive tumor microenvironment and enhance immunotherapy. Proc Natl Acad Sci U S A 109, 17561–17566 (2012). 23045683 10.1073/pnas.1215397109 PMC3491458

[14] Li B., Vascular endothelial growth factor blockade reduces intratumoral regulatory T cells and enhances the efficacy of a GM-CSF-secreting cancer immunotherapy. Clin Cancer Res 12, 6808–6816 (2006). 17121902 10.1158/1078-0432.CCR-06-1558

[15] Rahma O.E. & Hodi F.S. The Intersection between Tumor Angiogenesis and Immune Suppression. Clin Cancer Res 25, 5449–5457 (2019). 30944124 10.1158/1078-0432.CCR-18-1543

[16] Cakmak P., Spatial immune profiling defines a subset of human gliomas with functional tertiary lymphoid structures. Immunity 58, 2847–2863.e2848 (2025). 41125076 10.1016/j.immuni.2025.09.018

[17] Huang Y., Improving immune-vascular crosstalk for cancer immunotherapy. Nat Rev Immunol 18, 195–203 (2018). 29332937 10.1038/nri.2017.145 PMC5922422

[18] Cabrita R., Tertiary lymphoid structures improve immunotherapy and survival in melanoma. Nature 577, 561–565 (2020). 31942071 10.1038/s41586-019-1914-8

[19] Meylan M., Tertiary lymphoid structures generate and propagate anti-tumor antibody-producing plasma cells in renal cell cancer. Immunity 55, 527–541.e525 (2022). 35231421 10.1016/j.immuni.2022.02.001

[20] Laumont C.M., Banville A.C., Gilardi M., Hollern D.P. & Nelson B.H. Tumour-infiltrating B cells: immunological mechanisms, clinical impact and therapeutic opportunities. Nat Rev Cancer 22, 414–430 (2022). 35393541 10.1038/s41568-022-00466-1 PMC9678336

[21] Lü M.J.S., Repeated responders to bevacizumab combination treatment in recurrent glioblastoma: a retrospective case study. J Neurooncol 175, 869–878 (2025). 40668315 10.1007/s11060-025-05162-2 PMC12420756

[22] Parker S., Immunotoxin-αCD40 therapy activates innate and adaptive immunity and generates a durable antitumor response in glioblastoma models. Sci Transl Med 15, eabn5649 (2023). 36753564 10.1126/scitranslmed.abn5649 PMC10440725

[23] Madkouri R., Immune classifications with cytotoxic CD8(+) and Th17 infiltrates are predictors of clinical prognosis in glioblastoma. Oncoimmunology 6, e1321186 (2017). 28680758 10.1080/2162402X.2017.1321186 PMC5486170

[24] van de Walle T., Tertiary Lymphoid Structures in the Central Nervous System: Implications for Glioblastoma. Front Immunol 12, 724739 (2021). 34539661 10.3389/fimmu.2021.724739 PMC8442660

[25] Gu-Trantien C., CXCL13-producing TFH cells link immune suppression and adaptive memory in human breast cancer. JCI Insight 2(2017). 10.1172/jci.insight.91487 PMC5453706 28570278

[26] Gu-Trantien C., CD4^+^ follicular helper T cell infiltration predicts breast cancer survival. J Clin Invest 123, 2873–2892 (2013). 23778140 10.1172/JCI67428 PMC3696556

[27] Joshi N.S., Regulatory T Cells in Tumor-Associated Tertiary Lymphoid Structures Suppress Anti-tumor T Cell Responses. Immunity 43, 579–590 (2015). 26341400 10.1016/j.immuni.2015.08.006 PMC4826619

[28] van der Woude L.L., Gorris M.A.J., Halilovic A., Figdor C.G. & de Vries I.J.M. Migrating into the Tumor: a Roadmap for T Cells. Trends Cancer 3, 797–808 (2017). 29120755 10.1016/j.trecan.2017.09.006

[29] De Palma M., Biziato D. & Petrova T.V. Microenvironmental regulation of tumour angiogenesis. Nat Rev Cancer 17, 457–474 (2017). 28706266 10.1038/nrc.2017.51

[30] Lanitis E., Irving M. & Coukos G. Targeting the tumor vasculature to enhance T cell activity. Curr Opin Immunol 33, 55–63 (2015). 25665467 10.1016/j.coi.2015.01.011 PMC4896929

[31] Jain R.K. Normalizing tumor vasculature with anti-angiogenic therapy: a new paradigm for combination therapy. Nat Med 7, 987–989 (2001). 11533692 10.1038/nm0901-987

[32] Yadav K., Immunohistochemistry study of tumor vascular normalization and anti-angiogenic effects of sunitinib versus bevacizumab prior to dose-dense doxorubicin/cyclophosphamide chemotherapy in HER2-negative breast cancer. Breast Cancer Res Treat 192, 131–142 (2022). 34928481 10.1007/s10549-021-06470-7 PMC8841320

[33] Huang Y., Goel S., Duda D.G., Fukumura D. & Jain R.K. Vascular normalization as an emerging strategy to enhance cancer immunotherapy. Cancer Res 73, 2943–2948 (2013). 23440426 10.1158/0008-5472.CAN-12-4354 PMC3655127

[34] Morikawa S., Abnormalities in pericytes on blood vessels and endothelial sprouts in tumors. Am J Pathol 160, 985–1000 (2002). 11891196 10.1016/S0002-9440(10)64920-6 PMC1867175

[35] Winkler F., Kinetics of vascular normalization by VEGFR2 blockade governs brain tumor response to radiation: role of oxygenation, angiopoietin-1, and matrix metalloproteinases. Cancer Cell 6, 553–563 (2004). 15607960 10.1016/j.ccr.2004.10.011

[36] Payne L.B., The pericyte microenvironment during vascular development. Microcirculation 26, e12554 (2019). 31066166 10.1111/micc.12554 PMC6834874

[37] Takeuchi H., Hashimoto N., Kitai R., Kubota T. & Kikuta K. Proliferation of vascular smooth muscle cells in glioblastoma multiforme. J Neurosurg 113, 218–224 (2010). 19929197 10.3171/2009.10.JNS08631

[38] Martin J.D., Seano G. & Jain R.K. Normalizing Function of Tumor Vessels: Progress, Opportunities, and Challenges. Annu Rev Physiol 81, 505–534 (2019). 30742782 10.1146/annurev-physiol-020518-114700 PMC6571025

[39] Braun S., Pericytes orchestrate a tumor-restraining microenvironment in glioblastoma. Nat Commun 16, 10918 (2025). 41339360 10.1038/s41467-025-66985-1 PMC12680652

[40] Touat-Hamici Z., Role of lipid phosphate phosphatase 3 in human aortic endothelial cell function. Cardiovasc Res 112, 702–713 (2016). 27694435 10.1093/cvr/cvw217 PMC5157138

[41] Krüger K., Microvessel proliferation by co-expression of endothelial nestin and Ki-67 is associated with a basal-like phenotype and aggressive features in breast cancer. Breast 22, 282–288 (2013). 22840462 10.1016/j.breast.2012.07.008

[42] Hellwig S.M., Endothelial CD34 is suppressed in human malignancies: role of angiogenic factors. Cancer Lett 120, 203–211 (1997). 9461038 10.1016/s0304-3835(97)00310-8

[43] Song E., VEGF-C-driven lymphatic drainage enables immunosurveillance of brain tumours. Nature 577, 689–694 (2020). 31942068 10.1038/s41586-019-1912-x PMC7100608

[44] Man S., CXCL12-induced monocyte-endothelial interactions promote lymphocyte transmigration across an in vitro blood-brain barrier. Sci Transl Med 4, 119ra114 (2012). 10.1126/scitranslmed.3003197 PMC3710123 22301555

[45] Nourshargh S. & Alon R. Leukocyte migration into inflamed tissues. Immunity 41, 694–707 (2014). 25517612 10.1016/j.immuni.2014.10.008

[46] Masopust D., Guidelines for T cell nomenclature. Nat Rev Immunol (2025). 10.1038/s41577-025-01246-2 41254225

[47] Artis D. & Spits H. The biology of innate lymphoid cells. Nature 517, 293–301 (2015). 25592534 10.1038/nature14189

[48] Koprivica I., ILC3: a case of conflicted identity. Front Immunol 14, 1271699 (2023). 37915588 10.3389/fimmu.2023.1271699 PMC10616800

[49] Dean I., Rapid functional impairment of natural killer cells following tumor entry limits anti-tumor immunity. Nat Commun 15, 683 (2024). 38267402 10.1038/s41467-024-44789-z PMC10808449

[50] Arroyo-Díaz N.M., Interferon-γ production by Tfh cells is required for CXCR3(+) pre-memory B cell differentiation and subsequent lung-resident memory B cell responses. Immunity 56, 2358–2372.e2355 (2023). 37699392 10.1016/j.immuni.2023.08.015 PMC10592015

[51] Noël G., Functional Th1-oriented T follicular helper cells that infiltrate human breast cancer promote effective adaptive immunity. J Clin Invest 131(2021). 10.1172/JCI139905 PMC8483751 34411002

[52] Luo W., Weisel F. & Shlomchik M.J. B Cell Receptor and CD40 Signaling Are Rewired for Synergistic Induction of the c-Myc Transcription Factor in Germinal Center B Cells. Immunity 48, 313–326.e315 (2018). 29396161 10.1016/j.immuni.2018.01.008 PMC5821563

[53] Han S., Zheng B., Schatz D.G., Spanopoulou E. & Kelsoe G. Neoteny in lymphocytes: Rag1 and Rag2 expression in germinal center B cells. Science 274, 2094–2097 (1996). 8953043 10.1126/science.274.5295.2094

[54] Giachino C., Padovan E. & Lanzavecchia A. Re-expression of RAG-1 and RAG-2 genes and evidence for secondary rearrangements in human germinal center B lymphocytes. Eur J Immunol 28, 3506–3513 (1998). 9842893 10.1002/(SICI)1521-4141(199811)28:11<3506::AID-IMMU3506>3.0.CO;2-J

[55] Hillion S., Rochas C., Youinou P. & Jamin C. Signaling pathways regulating RAG expression in B lymphocytes. Autoimmun Rev 8, 599–604 (2009). 19393209 10.1016/j.autrev.2009.02.004

[56] Ochiai K., Transcriptional regulation of germinal center B and plasma cell fates by dynamical control of IRF4. Immunity 38, 918–929 (2013). 23684984 10.1016/j.immuni.2013.04.009 PMC3690549

[57] Li H., Mature tertiary lymphoid structures evoke intra-tumoral T and B cell responses via progenitor exhausted CD4(+) T cells in head and neck cancer. Nat Commun 16, 4228 (2025). 40335494 10.1038/s41467-025-59341-w PMC12059173

[58] Siliņa K., Germinal Centers Determine the Prognostic Relevance of Tertiary Lymphoid Structures and Are Impaired by Corticosteroids in Lung Squamous Cell Carcinoma. Cancer Res 78, 1308–1320 (2018). 29279354 10.1158/0008-5472.CAN-17-1987

[59] Vanhersecke L., Mature tertiary lymphoid structures predict immune checkpoint inhibitor efficacy in solid tumors independently of PD-L1 expression. Nat Cancer 2, 794–802 (2021). 35118423 10.1038/s43018-021-00232-6 PMC8809887

[60] Sofopoulos M., The prognostic significance of peritumoral tertiary lymphoid structures in breast cancer. Cancer Immunol Immunother 68, 1733–1745 (2019). 31598757 10.1007/s00262-019-02407-8 PMC11028375

[61] Hayashi Y., Density and maturity of peritumoral tertiary lymphoid structures in oesophageal squamous cell carcinoma predicts patient survival and response to immune checkpoint inhibitors. Br J Cancer 128, 2175–2185 (2023). 37016103 10.1038/s41416-023-02235-9 PMC10241865

[62] Lin Y.J., Wu C.Y., Wu J.Y. & Lim M. The Role of Myeloid Cells in GBM Immunosuppression. Front Immunol 13, 887781 (2022). 35711434 10.3389/fimmu.2022.887781 PMC9192945

[63] Kashyap A.S., Optimized antiangiogenic reprogramming of the tumor microenvironment potentiates CD40 immunotherapy. Proc Natl Acad Sci U S A 117, 541–551 (2020). 31889004 10.1073/pnas.1902145116 PMC6955310

[64] King E.M., Gpnmb and Spp1 mark a conserved macrophage injury response masking fibrosis-specific programming in the lung. JCI Insight 9(2024). 10.1172/jci.insight.182700 PMC11665561 39509324

[65] Zou B., A highly conserved host lipase deacylates oxidized phospholipids and ameliorates acute lung injury in mice. Elife 10(2021). 10.7554/eLife.70938 PMC8594946 34783310

[66] Chen Y., Macrophage CCL7 promotes resistance to immunotherapy for colorectal cancer by regulating the infiltration of macrophages and CD8(+) T cells. J Immunother Cancer 13(2025). 10.1136/jitc-2025-013027 PMC12645621 41290259

[67] Lanaya H., EGFR has a tumour-promoting role in liver macrophages during hepatocellular carcinoma formation. Nat Cell Biol 16, 972–977 (2014). 25173978 10.1038/ncb3031 PMC4183558

[68] Jung S.H., Spatiotemporal dynamics of macrophage heterogeneity and a potential function of Trem2(hi) macrophages in infarcted hearts. Nat Commun 13, 4580 (2022). 35933399 10.1038/s41467-022-32284-2 PMC9357004

[69] Huang Z., Integrated analyses of single-cell transcriptomics identify metastasis-associated myeloid subpopulations in breast cancer lung metastasis. Front Immunol 14, 1180402 (2023). 37483625 10.3389/fimmu.2023.1180402 PMC10361816

[70] Satoh J.I., Kino Y., Yanaizu M., Ishida T. & Saito Y. Microglia express TMEM119 in the brains of Nasu-Hakola disease. Intractable Rare Dis Res 8, 260–265 (2019). 31890453 10.5582/irdr.2019.01123 PMC6929589

[71] Cohen M.H., Shen Y.L., Keegan P. & Pazdur R. FDA drug approval summary: bevacizumab (Avastin) as treatment of recurrent glioblastoma multiforme. Oncologist 14, 1131–1138 (2009). 19897538 10.1634/theoncologist.2009-0121

[72] Gilbert M.R., A randomized trial of bevacizumab for newly diagnosed glioblastoma. N Engl J Med 370, 699–708 (2014). 24552317 10.1056/NEJMoa1308573 PMC4201043

[73] Jain R.K. Normalization of tumor vasculature: an emerging concept in antiangiogenic therapy. Science 307, 58–62 (2005). 15637262 10.1126/science.1104819

[74] Liu Z.L., Chen H.H., Zheng L.L., Sun L.P. & Shi L. Angiogenic signaling pathways and anti-angiogenic therapy for cancer. Signal Transduct Target Ther 8, 198 (2023). 37169756 10.1038/s41392-023-01460-1 PMC10175505

[75] Batchelor T.T., AZD2171, a pan-VEGF receptor tyrosine kinase inhibitor, normalizes tumor vasculature and alleviates edema in glioblastoma patients. Cancer Cell 11, 83–95 (2007). 17222792 10.1016/j.ccr.2006.11.021 PMC2748664

[76] Paez-Ribes M., Antiangiogenic therapy elicits malignant progression of tumors to increased local invasion and distant metastasis. Cancer Cell 15, 220–231 (2009). 19249680 10.1016/j.ccr.2009.01.027 PMC2874829

[77] Xie Y., Key molecular alterations in endothelial cells in human glioblastoma uncovered through single-cell RNA sequencing. JCI Insight 6(2021). 10.1172/jci.insight.150861 PMC8410070 34228647

[78] McEver R.P. & Zhu C. Rolling cell adhesion. Annu Rev Cell Dev Biol 26, 363–396 (2010). 19575676 10.1146/annurev.cellbio.042308.113238 PMC3557855

[79] Meylan M., Early Hepatic Lesions Display Immature Tertiary Lymphoid Structures and Show Elevated Expression of Immune Inhibitory and Immunosuppressive Molecules. Clin Cancer Res 26, 4381–4389 (2020). 32269054 10.1158/1078-0432.CCR-19-2929

[80] Cakmak P., Spatial immune profiling defines a subset of human gliomas with functional tertiary lymphoid structures. Immunity 58, 2847–2863 e2848 (2025). 41125076 10.1016/j.immuni.2025.09.018

[81] van Hooren L., Agonistic CD40 therapy induces tertiary lymphoid structures but impairs responses to checkpoint blockade in glioma. Nat Commun 12, 4127 (2021). 34226552 10.1038/s41467-021-24347-7 PMC8257767

[82] Zhou Q., Tertiary lymphoid structures in glioblastoma: Association with multiparametric MRI imaging phenotypic features and patient survival. Neurooncol Adv 8, vdaf263 (2026). 41727339 10.1093/noajnl/vdaf263 PMC12924633

[83] Mauldin I.S., Proliferating CD8(+) T Cell Infiltrates Are Associated with Improved Survival in Glioblastoma. Cells 10(2021). 10.3390/cells10123378 PMC8699921 34943886

[84] Lee-Chang C., Myeloid-Derived Suppressive Cells Promote B cell-Mediated Immunosuppression via Transfer of PD-L1 in Glioblastoma. Cancer Immunol Res 7, 1928–1943 (2019). 31530559 10.1158/2326-6066.CIR-19-0240 PMC6891201

[85] Iglesia M.D., Genomic Analysis of Immune Cell Infiltrates Across 11 Tumor Types. J Natl Cancer Inst 108(2016). 10.1093/jnci/djw144 PMC5241901 27335052

[86] Ramachandran M., Tailoring vascular phenotype through AAV therapy promotes anti-tumor immunity in glioma. Cancer Cell 41, 1134–1151 e1110 (2023). 37172581 10.1016/j.ccell.2023.04.010

[87] Osorio J.C., Fc-optimized CD40 agonistic antibody elicits tertiary lymphoid structure formation and systemic antitumor immunity in metastatic cancer. Cancer Cell 43, 1902–1916 e1909 (2025). 40816292 10.1016/j.ccell.2025.07.013 PMC12360484

[88] Phan T.G., High affinity germinal center B cells are actively selected into the plasma cell compartment. J Exp Med 203, 2419–2424 (2006). 17030950 10.1084/jem.20061254 PMC2118125

[89] Zhai C.C., Kinetics Evaluation of IgM and IgG Levels in the Mice Infected with Trichinella spiralis Experimentally Using ES Antigens from Different Developmental Stages of the Parasite. Iran J Parasitol 14, 223–230 (2019). 31543910 PMC6737358

[90] Woroniecka K., T-Cell Exhaustion Signatures Vary with Tumor Type and Are Severe in Glioblastoma. Clin Cancer Res 24, 4175–4186 (2018). 29437767 10.1158/1078-0432.CCR-17-1846 PMC6081269

[91] Chandramohan V., Construction of an immunotoxin, D2C7-(scdsFv)-PE38KDEL, targeting EGFRwt and EGFRvIII for brain tumor therapy. Clin Cancer Res 19, 4717–4727 (2013). 23857604 10.1158/1078-0432.CCR-12-3891 PMC3766439

[92] Urup T., Transcriptional changes induced by bevacizumab combination therapy in responding and non-responding recurrent glioblastoma patients. BMC Cancer 17, 278 (2017). 28420326 10.1186/s12885-017-3251-3 PMC5395849

[93] Newman A.M., Robust enumeration of cell subsets from tissue expression profiles. Nat Methods 12, 453–457 (2015). 25822800 10.1038/nmeth.3337 PMC4739640

[94] Li T., TIMER2.0 for analysis of tumor-infiltrating immune cells. Nucleic Acids Res 48, W509–W514 (2020). 32442275 10.1093/nar/gkaa407 PMC7319575

